# Integrative computational–experimental discovery and translation of antifungal peptides for multidrug-resistant fungi

**DOI:** 10.3389/fmicb.2026.1861239

**Published:** 2026-06-29

**Authors:** Lin Jiang, Shuting Yang, Yi Li, Yule Zhang, Xuejiao Ren, Wei Liu, Hongxun Fu, Changjie Bao, Qi Qi, Tianhui Liu, Meiyan Sun, Xianzhen Li

**Affiliations:** 1 School of Biological Engineering, Dalian Polytechnic University, Dalian, China; 2 College of Laboratory Medicine, Jilin Medical University, Jilin, China; 3 School of Life Science and Technology, Changchun University of Science and Technology, Changchun, China

**Keywords:** antifungal peptides, *Candida auris*, drug delivery systems, machine learning, molecular modeling, multidrug-resistant fungi, peptide design, translational medicine

## Abstract

Multidrug-resistant fungal infections caused by *Candida* and *Aspergillus* species have become one of the major global health concerns, especially among immunocompromised individuals. The small number of antifungals available and the rapid emergence of resistance to azoles, echinocandins and polyenes underscore the urgent need to develop alternative therapeutic strategies with different mechanisms of action. Antifungal peptides (AFPs) have attracted increasing attention as promising candidates due to their broad-spectrum activity, multimodal mechanisms of action, and their low likelihood of resistance development. This review presents a thorough and holistic summary of the research on AFPs that target clinically significant drug-resistant fungi such as *Candida auris*, azole-resistant *Candida albicans*, and triazole-resistant *Aspergillus fumigatus*. We review the structural and physicochemical properties of AFPs and address their various antifungal mechanisms, which include membrane disruption, oxidative stress induction, and disruption of intracellular homeostasis, as well as biofilm inhibition. We further highlight an emerging computational–experimental pipeline to discover and optimize AFPs, combining sequence mining, machine learning-based screening, molecular docking, molecular dynamics simulations, and *in vitro* and *in vivo* validation. We also explore the major translational challenges, such as hemolytic toxicity, proteolytic instability, pharmacokinetic constraints, manufacturing complexity, regulatory concerns, and sustainable peptide manufacturing strategies, and discuss advanced delivery systems (e.g., liposomes, PLGA nanoparticles, chitosan-based systems, and hydrogels) to improve therapeutic efficacy and stability. In summary, this review proposes an integrated translational development framework that connects computational design, experimental validation, and delivery engineering, thereby positioning AFPs as a promising next-generation strategy in the fight against multidrug-resistant fungal infections.

## 1 Introduction

The problem of invasive fungal infections (IFIs) has become one of the most significant public health concerns around the world. The latest estimates show that invasive fungal infections affect about 6.5 million people every year and the mortality rate remains unacceptably high (Denning, 2024). IFIs are more likely to impact immunocompromised people and those who suffer from chronic underlying conditions (Casalini et al., 2024). The diagnosis of fungal infections is frequently challenging due to non-specific clinical presentations and poor diagnostic sensitivity. The increasing emergence of antifungal resistance further complicates disease management and therapeutic outcomes. To address the growing threat posed by fungal pathogens, the World Health Organization (WHO) published the first Fungal Priority Pathogens List (FPPL) in 2022 (WHO, 2022). The FPPL categorizes 19 fungal pathogens into critical, high-, and medium-priority groups based on their public health significance and unmet clinical needs (Casalini et al., 2024; WHO, 2022). The initiative aims to facilitate research prioritization, surveillance, and therapeutic development for clinically significant fungal pathogens.

*Candida auris* (*C. auris*), *Aspergillus fumigatus*, and *Candida albicans* are classified within the FPPL critical-priority group due to their major clinical impact, increasing antifungal resistance, and limited therapeutic options. Antifungal resistance was one of the major weighted criteria used in the WHO FPPL prioritization framework and underlines the criticality of developing new antifungal approaches (WHO, 2022). Among these pathogens, *C. auris* has attracted substantial attention because of its environmental persistence, rapid nosocomial transmission, and multidrug-resistant phenotype, which together pose significant challenges to infection control worldwide (Calvo et al., 2024). Recent phylogenetic studies have proposed reclassification of *Candida auris* as *Candidozyma auris*, however, the term *C. auris* remains widely used in current clinical and antifungal research literature (Salmanton-Garcia et al., 2026). Moreover, *C. auris* readily forms biofilms, and frequently exhibits resistance to multiple classes of antifungal agents, thereby limiting available therapeutic options (Jones et al., 2024). A new meta-analysis comprising 81 studies indicated that *C. auris* exhibits high resistance rates to fluconazole (92.5%), voriconazole (49.0%), and amphotericin B (51.0%), which highlights the global challenge of antifungal resistance (Achilonu et al., 2025). These constraints underscore the urgent need to develop alternative antifungal agents that are more effective, less toxic, and have a lower likelihood of developing resistance.

Mechanisms underlying antifungal resistance include target-site mutations, efflux pump overexpression, biofilm formation, stress adaptation pathways, and persistence-associated phenotypes (Haq et al., 2025). The development of resistance can be explained in part by the shortage of antifungal drug classes, as well as the relatively conserved mechanisms of action of those drugs (Du et al., 2025). Antifungal peptides (AFPs) are an extremely diverse family of naturally occurring, synthetic, and computationally generated peptides that, as opposed to standard antifungal drugs, frequently display antifungal properties through multiple overlapping mechanisms of action (Fernandez de Ullivarri et al., 2020; Li et al., 2021). AFPs are generally amphiphilic in nature and positively charged, which allows them to disrupt fungal cell membranes through electrostatic interactions, penetrate fungal cells to disrupt intracellular targets, or exhibit indirect antifungal activity through immunomodulatory mechanisms (Yang et al., 2020). These multimodal mechanisms are considered less likely to induce stable resistance as compared with traditional antifungal agents (Li et al., 2021). The development of AFP is challenging due to the structural similarities of mammalian and fungal cells which can increase the risk of toxicity and limit selective targeting. Additionally, the complex fungal cell wall and biofilm-associated phenotypes can reduce the penetration of peptides and reduce their antifungal efficacy. AFPs have been identified across different biological kingdoms, including animals, plants, and microorganisms. In addition to naturally occurring AFPs, the development of computationally designed peptides, hybrid “Franken-peptides,” and peptide–drug conjugates with better antifungal selectivity and physicochemical properties have been made possible through advanced computational peptide engineering and synthetic biology. Despite the progress made in computational peptide engineering, the discovery and optimization of AFPs is still labor-intensive. Peptide activity, selectivity, and toxicity continue to require extensive *in vitro* and *in vivo* testing for further validation (Fernandez de Ullivarri et al., 2020).

AI and computational modeling have facilitated AFP screening and design workflows. New computational workflows, combined with high-throughput peptide synthesis and testing, have substantially accelerated AFP discovery and optimization cycles. During AFP development, sequence–activity relationship analysis can be performed more efficiently. Computational methods, including sequence mining, machine learning, molecular docking, and molecular dynamics, are increasingly used to predict antifungal activity of peptides, toxicity, and interactions with biological targets (Goles et al., 2024). In addition, many existing AFP prediction models have been adapted from antibacterial peptide datasets. Thus, they likely do not adequately capture fungal-specific biological properties, thereby limiting prediction generalizability. However, despite the limitations of current computational models, predictive performance and translational applicability can be improved through incorporation of fungal-specific datasets and expanded experimental validation (Wang S. et al., 2025; Wang Y. et al., 2025).

This review highlights structural features, mechanisms of action, developments in computational design, and potential applications of AFPs in fighting multidrug-resistant fungal pathogens. Particular emphasis is placed on current challenges in AFP development, including peptide stability, toxicity, and barriers to clinical translation, and particularly limitations related to dataset quality and model generalizability. We also analyze modern computation-assisted design strategies, and discuss both their potential and current limitations in AFP development.

## 2 Overview of natural AFPs

### 2.1 Classification and biological sources of AFPs

Table 1 shows that AFPs can be broadly classified into four primary groups according to their biological origins, including those derived from microorganisms, plants, invertebrates, and vertebrates. AFPs derived from different biological sources demonstrate notable diversity in their structural features, physicochemical traits, and fungal targeting properties (Huber et al., 2018). Nevertheless, most AFPs display broad-spectrum antifungal activity against clinically relevant fungi, including *Candida*, *Aspergillus*, and *Cryptococcus* species.

(1) Microbial-derived AFPs are generally associated with ecological niche competition and environmental adaptation and are secreted extracellularly by both prokaryotic organisms (bacteria and archaea) and eukaryotic fungi (Gong et al., 2024). Representative microbial AFPs, including archaeal peptides such as VLL-28, cyclic lipopeptides like Iturin A, and cysteine-rich fungal AFPs such as PgAFP, generally exhibit potent membrane-targeting activity and broad antifungal spectra, although their cytotoxicity and stability profiles vary among peptide classes (Huber et al., 2018; Klich et al., 1991; Notomista et al., 2015; Rodriguez-Martin et al., 2010; Roscetto et al., 2018). VLL-28 is the first archaeal antifungal peptide reported to exhibit antimicrobial activity against the genus *Candida*. Studies have shown that VLL-28 displays antimicrobial activity against ten clinically isolated *Candida* strains, with marked variation in potency; the most pronounced activity was observed against *Candida albicans* and *Candida parapsilosis* (Roscetto et al., 2018). Iturin A, produced by the *Bacillus subtilis* strain B-3, is a cyclic lipopeptide and exhibits strong antifungal activity against *Aspergillus* spp., *Fusarium* spp., and *Penicillium* spp. (Klich et al., 1991). PgAFP, derived from *Penicillium chrysogenum*, adopts a compact β-sheet structure and exhibits antimicrobial activity against filamentous fungi (*A. fumigatus*, *A. niger*, *A. terreus*, *N. crassa*, *P. chrysogenum*, *T. rubrum*) as well as yeasts (*C. albicans* and *S. cerevisiae*)(Huber et al., 2018).
(2) Plant-derived AFPs are key effector molecules of the plant innate immune and defense system (Gong et al., 2024). They can be induced upon pathogen infection or wounding, and can also be constitutively expressed in specific tissues to maintain basal defense (Chiu et al., 2022). Based on their sequences, cysteine residues, and functions, plant AFPs can be classified into chitinases, defensins, and snakins, as well as hevein-type and glycine-rich peptides (Tam et al., 2015). Chitinases are a representative class of plant-derived AFPs. For example, PvD1, which was isolated and characterized from common bean (*Phaseolus vulgaris*) seeds, exhibits broad-spectrum antimicrobial activity against members of the genera *Candida*, *Kluyveromyces*, *Saccharomyces*, *Fusarium*, and *Rhizoctonia* (Mello et al., 2011). The defensin DM-AMP1 shows inhibitory activity against multiple fungi in the presence of CaCl₂ and KCl₂(Osborn et al., 1995). In addition, SmAMP3, isolated and characterized from the leaves of *Stellaria media* L., is a basic, cysteine-rich peptide with potent antifungal activity against several important plant pathogens (Rogozhin et al., 2015) (see Table 1 for more details).
(3) Invertebrate-derived AFPs can be categorized by origin into those from marine invertebrates, insects, and other arthropods. Cm-p1 from the marine snail *Cenchritis muricatus* may contain a single hydrophilic α-helix structure (Lopez-Abarrategui et al., 2012). It exhibits no toxicity to mammalian cells and inhibits the growth of yeast and filamentous fungi (Lopez-Abarrategui et al., 2012). Zhang et al. isolated and identified an antifungal peptide named blapstin from the Chinese medicinal beetle *Blaps rhynchopetera*, which exhibits antifungal activity against *Candida albicans* (MIC = 7 μM) and *Trichophyton rubrum* (MIC = 5.3 μM) (Zhang et al., 2023). A small-sized peptide, gomesin, purified from the hemocytes of the wolf spider *Acanthoscurria gomesiana*, strongly inhibits the development of filamentous fungi and yeasts (Silva et al., 2000).
(4) Vertebrate-derived AFPs primarily originate from the adaptive immune system, representing antimicrobial peptides (AMPs) produced in response to pathogen attacks. Piscidins synthesized in the skin, respiratory, and digestive systems of various teleost species exhibit antimicrobial activity against fungi (such as *Candida albicans*) through membrane disruption mechanisms (Asensio-Calavia et al., 2023). Temporin G (TG), derived from amphibian frog skin, exhibits significant antifungal activity against *Candida* species, *Cryptococcus neoformans*, and *Aspergillus* species (D'Auria et al., 2022). It also effectively inhibits biofilm formation and reduces metabolic activity in mature biofilm cells (D'Auria et al., 2022). Mammalian AFPs consist of defensins, neutrophil-derived peptides, histones, and lactoferrin (Gong et al., 2024). Human defensins exhibit antifungal activity against *Candida* species. LL-37 exerts antifungal activity against *Candida albicans* through multiple mechanisms, including disruption of cell wall integrity, membrane permeabilization, and intracellular effects (Scarsini et al., 2015). Lactoferricin B inhibits the synergistic interaction of biofilms formed by three fungal strains (*Aspergillus fumigatus*, *Fusarium solani*, and *Candida albicans*) associated with keratitis (Sengupta et al., 2012).

**Table 1:** Overview of the sources, structural features, physicochemical properties, and mechanisms of action of AFPs.

Sources	Classification	Example	Structure	Physicochemical properties	Mechanisms of action	References
Microorganism	Archaea	VLL-28 (from *Sulfolobus islandicus*)	α-helix	Strongly cationic; net charge~ +10; hydrophobic residues ~35%	Membrane targeting; anti-biofilm vs. *Candida* clinical isolates	Notomista et al. (2015), Roscetto et al. (2018)
	Bacteria	Iturin A (from *Bacillus subtilis*)	Cyclic peptidolipid	Strongly amphipathic/hydrophobic; may show hemolytic/cytotoxic tendency at higher doses	Membrane permeabilization/lysis via lipid interaction	Klich et al. (1991)
	Fungi	PgAFP (from *Penicillium chrysogenum*)	β-sheet	Cationic, cysteine-rich, disulfide-stabilized β-fold; net charge ~ +4; hydrophobic residues ~27%	Cell-surface binding → membrane dysfunction → growth inhibition	Huber et al. (2018), Rodriguez-Martin et al. (2010)
Plants	Chitinases	PvD1 (from *Phaseolus vulgaris*)	Bridge	Disulfide-bridged, cysteine-rich fold; net charge ~ −1; hydrophobic residues ~21%	Plasma membrane permeabilization; inhibition of medium acidification; induction of intracellular ROS in fungi	Mello et al. (2011)
	Defensins	Dm-AMP1 (from *Dahlia merckii*)	Combined α-helix/β-sheet	Disulfide-stabilized; moderately cationic; net charge ~+2; hydrophobic residues ~35%	Targets fungal membrane lipids → permeabilization/ion imbalance	Osborn et al. (1995)
	Hevein-type	SmAMP3 (from *Stellaria media L.*)	Bridge	Basic; cysteine-rich; disulfide-bridged; net charge ~ +2; hydrophobic residues ~34%	Binds cell-wall-associated polysaccharides (chitin-related) and perturbs cell envelope integrity, resulting in growth inhibition	Rogozhin et al. (2015)
	Snakins	Snakin-1 (from *Solanum tuberosum*)	α-helix	Highly cationic, cysteine-rich (disulfide-stabilized); net charge ~ +8; hydrophobic residues ~31%	Non-membrane permeability-dependent, synergistic/additive enhancement of antifungal activity with defensins Targets fungus-specific molecules (non-membrane structures)	Segura et al. (1999)
	Gly-rich peptides	Cc-GRP (from *Coffea canephora*)	Gly-rich	Hydrophilic; net charge ~ −1; hydrophobic residues ~0%	Membrane permeabilization with intracellular/nuclear localization in fungal cells	Zottich et al. (2013)
Invertebrates	Marine invertebrate	Cm-p1 (from *Cenchritis muricatus*)	α-helix	Hydrophilic; low toxicity; mildly cationic	Growth inhibition via membrane interaction / permeabilization against yeasts and filamentous fungi	Lopez-Abarrategui et al. (2012)
	Insect	Blapstin (from *Blaps rhynchopetera*)	Bridge	Disulfide-bridged; low hemolysis/toxicity; net charge ~ +4; hydrophobic residues ~27%	Membrane lysis/permeabilization; inhibits *C. albicans* biofilm activity	Zhang et al. (2023)
	Other Arthropoda	Gomesin (from *Acanthoscurria gomesiana*)	β-sheet	Cationic, cysteine-rich; net charge ~ +6; hydrophobic residues ~33%	Rapid membrane permeabilization (pore-like)	Silva et al. (2000)
Vertebrates	Fish	Piscidins family (from Various fish taxa)	α-helix	Cationic, amphipathic α-helical peptides	Membrane disruption/permeabilization; broad antifungal activity	Asensio-Calavia et al. (2023)
	Amphibian	Temporins G (from *Rana temporaria*)	–	Short, hydrophobic peptide; mildly cationic; net charge ~ +2; hydrophobic residues ~61%	Moderate membrane perturbation; reduces *C. albicans* virulence traits and metabolic activity	D'Auria et al. (2022)
	Mammal	LL-37 (from *Homo sapiens; Pan troglodytes*)	α-helix	Cationic, amphipathic α-helix; net charge ~ +6; hydrophobic residues ~35%	Membrane permeabilization; anti-adhesion/anti-biofilm effects against *Candida* spp.	Scarsini et al. (2015)
		Lactoferricin B (from *Bos taurus*)	β-sheet	Cationic peptide with β-structure tendency; net charge ~ +8; hydrophobic residues ~48%	Anti-biofilm vs. keratitis-associated fungal pathogens	Sengupta et al. (2012)

### 2.2 Structural features and physicochemical properties

**Figure 1:**
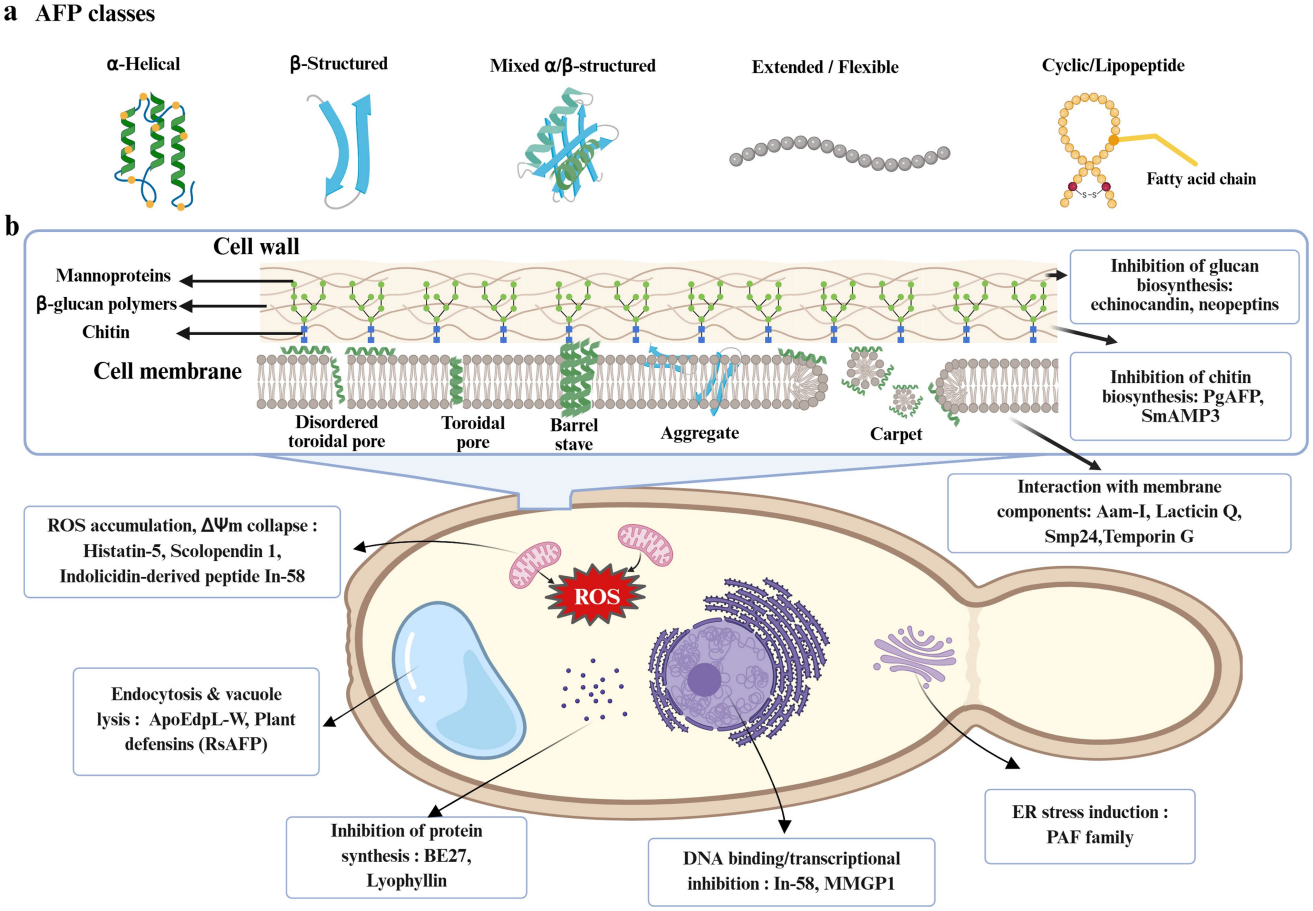
Structural classes and multilevel antifungal mechanisms of AFPs. AFPs can be classified into five major structural types: α-helical, β-structured, mixed α/β-structured, extended/flexible, and cyclic/lipopeptide forms **(a)**. These structural classes determine their physicochemical properties and antifungal selectivity. AFPs act through multi-tiered mechanisms targeting the fungal cell wall, cell membrane, and intracellular components **(b)**. The upper panel illustrates different models of membrane and cell wall interactions, including toroidal pore, barrel-stave, aggregate, and carpet models, as well as inhibition of glucan or chitin biosynthesis. The lower panel depicts intracellular antifungal mechanisms, such as reactive oxygen species (ROS) accumulation, mitochondrial membrane potential (ΔΨm) collapse, endocytosis and vacuole lysis, inhibition of DNA transcription or protein synthesis, and endoplasmic reticulum (ER) stress induction. Together, these multimodal actions underlie the potent antifungal efficacy and low resistance tendency of AFPs.

AFPs exhibit diverse structural conformations which significantly influence their biological activities and antifungal selectivity. As shown in Figure 1A, these include α-helical, β-structured, mixed α-elements and β-elements, extended/flexible, and cyclic or lipopeptide conformations (Oliveira Junior et al., 2025).

Extended or flexible AFPs generally lack stable secondary structures and are enriched in residues such as glycine and tryptophan, which confer considerable structural flexibility (Hsu et al., 2005). These peptides can interact with fungal membranes to form transient pores, penetrate fungal cells, and interfere with intracellular processes such as DNA replication and protein synthesis (Sneideris et al., 2023). α-Helical AFPs typically exhibit amphiphilic architectures characterized by spatial separation of hydrophilic and hydrophobic surfaces (Huang et al., 2010). Owing to their cationic nature, these peptides preferentially interact with anionic phospholipids in fungal membranes through electrostatic interactions (Sato and Feix, 2006). β-Structured AFPs are typically stabilized by multiple disulfide bonds that maintain their β-sheet-rich conformations. The structure also shows amphiphilic characteristics, with its ability to interact with the fungal cell membrane (Mani et al., 2006). Research shows that this conformation has an overall positive charge, and it forms non-covalent bonds with either β-glucans or chitin in fungal cell walls (Datta et al., 1996). Mixed α-helical and β-strand AFPs combine the flexibility of α-helices with the stability of β-strand disulfide bonds. Consequently, these AFPs may exert antifungal activity through both membrane-associated interactions and intracellular targeting mechanisms (Zasloff, 2002). Lipopeptidic or cyclic AFPs generally exhibit enhanced resistance to proteolytic degradation and improved thermal stability (Zhao et al., 2022). Certain AFPs contain stable covalent linkages such as ester bonds, thioether bonds, or disulfide bonds which contribute to cyclic or partially cyclic conformations (Oliveira Junior et al., 2025). In summary, the secondary structures of AFPs largely determine their physicochemical properties, including charge, amphiphilicity, and hydrophobicity. These physicochemical properties collectively influence antifungal activity, target selectivity, and translational applicability.

## 3 Mechanisms of antifungal action and resistance escape

APFs have a multitargeted and multimodal synergistic effect in working, and therefore it is hard to resist it (Zhang et al., 2021). The mode has very important benefits over the multidrug-resistant fungi. Figure 1B is a summary of the various actions and targets of AFPs.

### 3.1 Inhibition of cell wall component biosynthesis

The major building blocks of the cell wall in fungi are chitin, glucan, mannan and glycoproteins (Bowman and Free, 2006). This is an essential barrier to external environmental elements. These elements form a dynamic structure that protects the cell from osmotic pressure, external environmental stresses, and immune system attacks.

Chitin constitutes a minor (1–10%) yet crucial component of fungal cell walls (Bowman and Free, 2006). It forms cross-links within the β-glucan network and exhibits significant tensile strength, playing a key role in maintaining cell wall integrity. Disruption of chitin synthesis leads to fungal cell morphological distortion and increased cell wall permeability (Specht et al., 1996). Table 1 shows the AFPs. *Penicillium chrysogenum* Antifungal Protein (PgAFP) exhibits cationic properties. It significantly disrupts the chitin and β-glucan networks while altering the charge environment of the cell wall, thereby destabilizing the *Candida* cell wall (Huber et al., 2018). SmAMP3, derived from *Stellaria media L.*, specifically binds to fungal cell wall chitin, blocking fungal cell wall synthesis and repair, thereby reducing the mechanical strength of the cell wall. Studies show that SmAMP3 can significantly inhibit fungal spore germination and hyphal elongation at micromolar concentrations (IC₅₀ = 1.6–5.4 μM) (Rogozhin et al., 2015). β-1,3-glucan is a major structural component of the fungal cell wall. Echinocandins inhibit the synthesis of β-1,3-D-glucan in the fungal cell wall and exhibit potent activity against pathogens such as *Candida* and *Aspergillus* (Thorn et al., 2024). It has also been reported that neopeptins similarly inhibit β-1,3-glucan synthesis by interfering with fungal cell wall biosynthesis (Gong et al., 2024). Fungal cell wall proteins are predominantly glycoproteins, whose functions span multiple aspects of cellular homeostasis and physiological activities. These include maintaining cell morphology, regulating cell adhesion and migration/fusion, serving as a physical barrier against foreign invaders, modulating molecular transport and absorption, mediating intracellular signaling from external cues, and participating in cell wall synthesis and dynamic remodeling (Bowman and Free, 2006). Research has found that LL-37 can regulate the adhesion function of *Candida albicans* cell walls by upregulating the β-1,3-glucanase activity of the fungal Xog1p protein (Scarsini et al., 2015). Some AFPs do not directly target the fungal cell wall but act as cofactors to enhance the fungicidal potency of AMPs. For example, heat shock proteins in the *Candida albicans* cell wall promote the specific binding of Histatin 5 to β-1,3-glucan in the fungal cell wall (Jang et al., 2010). Subsequently, Histatin 5 induces intracellular ion imbalance, ultimately leading to fungal cell death.

### 3.2 Mechanisms of membrane disruption and permeabilization

AFPs act rapidly on fungal cell membranes and exhibit inhibitory and fungicidal activities at the peptide–membrane interface. Most of the membrane-directed AFPs do not act like conventional small-molecule antibiotics but rather utilize their amphiphilic and other biophysical properties to interact with the fungal cell membranes. These features allow electrostatic attachment, which in turn increases or aggregates in localized areas of membranes (Oliveira Junior et al., 2025). The local coverage threshold is reached, causing damage to the cell membrane, and changes in the arrangement of the lipids and permeability, resulting in the loss of cellular contents. At such a level, AFP can also show various modes of action (Figure 1B) which would affect membrane integrity.

The aggregation model constructs nonspecific channels on the cell surface of the fungus cell membranes which are randomly sized and in length but helps to facilitate the transportation of peptides across the bilayer (Wu et al., 1999). Simultaneously, peptides go through the reconfiguration of their conformational structure, passing into the membrane by way of micelle-like complexes. This system allows the AFPs to control fungal cell membrane permeability. E.g., Peptide Polymerase (I) - (PM1). In the barrel-stave model, the AFPs are binding with the lipid membrane. They become vertically embedded within the membrane and undergo polymerization, forming transmembrane pores that lead to the leakage of cellular contents and cell death. For example, Antiamoebin I (Aam-I) (Shenkarev et al., 2013). In the toroidal pore model, AFPs can embed into the hydrophobic core of the cell membrane, causing the phospholipid bilayer to bend inward and randomly form mixed cavities, ultimately leading to cell death. An example is Lacticin Q (Yoneyama et al., 2009). Randomly formed disordered toroidal pores may also occur (Bechinger, 1997). A classic example is the venom peptide Smp24 from Scorpio maurus palmatus (Bertelsen et al., 2023). Similarly to other models, charge adsorption initially occurs in the carpet model. Upon reaching a threshold, it alters membrane permeability, causing membrane disruption in a detergent-like manner. An example is Temporin G (derived from *Rana temporaria*) as shown in Table 1 (D'Auria et al., 2022). The membrane activity of AFPs cannot be simplified to membrane disruption alone, as AFPs likely interact with multiple physiological processes within fungal cells. Overall, these membrane-targeting mechanisms demonstrate the role of the physicochemical properties of AFPs, including charge, amphiphilicity, and conformational flexibility.

### 3.3 Interference with intracellular targets and key physiological processes

Some AFPs can penetrate fungal membranes and interact with intracellular targets (Figure 1B). As described above, Histatin 5 exhibits a multi-target antifungal mechanism. It initially binds to β-1,3-glucan in the cell wall of *Candida albicans*. It then translocates into the cytoplasm, where it targets mitochondria and disrupts ATP metabolism. This leads to ion imbalance and ultimately fungal cell death (Jang et al., 2010). For example, the indolicidin-derived peptide In-58 disrupts membrane permeability, enters fungal cells, and interacts with DNA, thereby affecting nucleic acid processes (Li et al., 2024). Simultaneously, it reduces the mitochondrial membrane potential (ΔΨm) in fungi, induces the production and accumulation of reactive oxygen species (ROS), thereby activating stress response pathways and ultimately inhibiting fungal viability. Scolopendin 1, significantly elevates intracellular Ca^2+^ concentration while inducing ROS accumulation in *Candida albicans* cells (Choi et al., 2014). This promotes the release of the apoptotic factor cytochrome C, thereby activating the apoptosis pathway. AFPs (ApoEdpL-W) derived from human apolipoprotein E bind to the cytoplasmic membrane of *Candida albicans*, are transported via endocytosis to vacuoles, and subsequently accumulate (Rossignol et al., 2011). From *Beta vulgaris*, BE27 exhibits typical RNA N-glycosylase activity, cleaving specific sites on fungal 28S rRNA to inhibit protein synthesis and induce cell death (Citores et al., 2016). Lyophyllin as an antifungal ribosomal inactivating protein (RIP) also attacks the ribosome mechanism by inhibiting elongation factor binding to stop protein synthesis (Lam and Ng, 2001). A marine metagenome-derived peptide (MMGP1) has a strong binding tendency with fungal DNA and forms peptide-DNA complexes. It inhibits the synthesis of mRNA and causes the accumulation of endogenous reactive oxygen species (ROS) (Pushpanathan et al., 2013). The Penicillium antifungal protein (PAF) family is able to trigger and activate the pathway of unfolded protein response (UPR) signaling to promote endoplasmic reticulum stress and thus inhibit fungus growth (Richie et al., 2009).

### 3.4 Immunomodulation and biofilm disruption mechanisms

AFPs disrupt fungal biofilms and modulate host immune responses against fungal pathogens. S100A12, a human AFP, binds to and penetrates fungal membranes, disturbing membrane structures and leading to the production of reactive oxygen species (ROS) (Roy et al., 2024). It can limit biofilm formation in *Fusarium* species and improve fungal clearance in infection models by enhancing macrophage phagocytosis (Roy et al., 2024). The polysaccharide capsule is a key virulence factor in *Cryptococcus neoformans*, contributing to immune evasion. Glucuronoxylomannan (GXM) can bind to SP1, disrupting capsule integrity (Liu et al., 2023). This structure encapsulates fungal cells and prevents macrophage recognition and phagocytosis, and also contributes to biofilm formation (Liu et al., 2023). AFPs can prevent biofilm formation by inhibiting adhesion, decreasing extracellular polymeric substance (EPS) production, and disrupting established biofilms. WMR peptides can suppress *Candida* biofilm formation and disrupt mature biofilms at sub-MIC levels by downregulating efflux pump genes (e.g., CDR1, MDR1) and inducing intracellular oxidative stress (Maione et al., 2022). Derived from snake venom, the peptide fragment Ctn[15–34] disrupts fungal membranes and the structural integrity of the biofilm matrix, resulting in membrane potential collapse and fungal cell death (Aguiar et al., 2020). AFPs not only exert direct antifungal effects but also modulate host immune responses, which may enhance their therapeutic potential against biofilm-associated fungal infections. These findings suggest that AFPs exert antifungal activity through dual mechanisms by directly disrupting biofilms while indirectly enhancing host immune responses against persistent fungal infections.

### 3.5 Anti-resistance properties and resistance escape mechanisms

Biofilm formation, active efflux pumps, and antioxidant defense systems contribute to fungal resistance against conventional antifungal agents. AFPs act through multiple highly efficient mechanisms, whereas stable antifungal resistance against AFPs remains relatively uncommon. Resistance mediated by single-target mutations is also relatively uncommon. This is evident in the WMR peptide, which downregulates some efflux pump genes (*CDR1, MDR1*) thereby enhancing antifungal efficacy (Maione et al., 2022). Likewise, GmAMP inhibits biofilm formation and alters membrane potential in fluconazole-resistant *Candida tropicalis* (Cai et al., 2025). GmAMP also enhances antifungal activity by promoting ROS accumulation and disrupting antioxidant defenses. In addition, AFPs can interfere with fungal stress response pathways. The peptide ToAP2 inhibits filamentation and shows synergistic effects with amphotericin B and fluconazole (do Nascimento Dias et al., 2020). Biofilm structures further limit drug penetration and immune clearance. AFPs can overcome this barrier by disrupting extracellular matrix components and downregulating quorum-sensing genes (e.g., *EFG1, HWP1*) (Mathe and Van Dijck, 2013). The multi-target and multimodal nature of AFPs enables them to circumvent resistance associated with target mutations. They also modulate stress response pathways to inhibit resistance development. These features highlight the potential of AFPs as promising candidates for addressing antifungal resistance. Overall, the ability of AFPs to target multiple pathways and interfere with resistance mechanisms positions them as a promising strategy for overcoming antifungal drug resistance.

## 4 *In silico* screening and optimization pipeline

**Figure 2:**
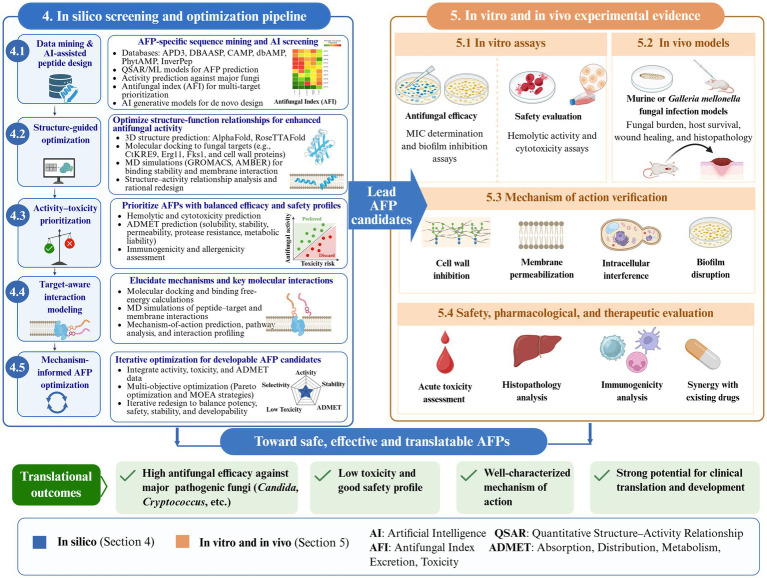
Integrated computational–experimental development pipeline of antifungal peptides (AFPs) against multidrug-resistant fungal pathogens. Computational optimization and experimental validation workflow for antifungal peptide (AFP) development. The *in silico* pipeline includes AI-assisted peptide design, structure-guided optimization, activity–toxicity prioritization, target interaction modeling, and mechanism-informed AFP refinement. Experimental validation includes *in vitro* antifungal activity and biosafety assays, mechanism-of-action studies, *in vivo* infection models, and therapeutic evaluation of AFP candidates against multidrug-resistant fungal pathogens.

AFPs have emerged as promising therapeutic candidates for combating multidrug-resistant fungal infections. Despite the increasing interest in peptide-based anti-infective therapeutics, only a limited number of antimicrobial or antifungal peptide-based agents have successfully advanced into clinical development or received regulatory approval, highlighting the persistent translational challenges associated with peptide therapeutics (Al Musaimi, 2025). Traditionally, the discovery and optimization of AFPs have depended on time-consuming experimental screening approaches such as peptide isolation from natural sources, activity-guided purification, and systematic antimicrobial assays. These methods are often limited by poor scalability and low screening efficiency, and are often unable to simultaneously optimize antifungal activity, selectivity, toxicity, and developability. Recent advances in artificial intelligence (AI), molecular modeling, and high-performance computing have substantially reshaped AFP discovery strategies by enabling computational sequence mining, structure-guided optimization, activity–toxicity prioritization, and mechanistic interpretation. The integrated computational–experimental development framework discussed in this review is summarized in Figure 2.

### 4.1 Computational screening and prioritization of AFP candidates

Advances in genomics, transcriptomics, and proteomics have generated large-scale biological datasets that provide important resources for AFP discovery and computational screening. The PhytAMP and InverPep databases are domain-specific databases that store AMPs of plants and invertebrates, respectively (Dijksteel et al., 2021; Gomez et al., 2017). Additionally, there are larger general-purpose peptide databases such as APD3 that integrate experimentally validated peptide sequences, physicochemical descriptors, and biological activity annotations for peptide discovery and rational design (Wang et al., 2016). The Collection of Anti-Microbial Peptides (CAMP) further supports AMP activity prediction and peptide annotation (Waghu et al., 2016). dbAMP further enables direct identification of putative AMPs from genomic and proteomic sequencing datasets (Jhong et al., 2022). The DBAASP database further supports *de novo* AMP design through peptide structure prediction and biocompatibility evaluation (Pirtskhalava et al., 2021). Together, these databases provide important computational foundations for peptide sequence mining, activity prediction, physicochemical characterization, and structural annotation.

Peptide discovery has been greatly advanced by the use of AMP-focused databases and computational frameworks. However, most rely on general antibacterial AMP datasets, which makes them difficult to directly apply to AFP screening. Fungal pathogens have unique characteristics such as distinct membrane compositions, resistance mechanisms and intracellular targets that are often not present in many general AMP datasets. These limitations point to the need for AFP-focused computational prioritization approaches that jointly consider antifungal activity, fungal selectivity, toxicity, and developability (Zhang et al., 2022).

QSAR models were among the earliest AI-powered approaches to AMP design in computational peptide screening (Hilpert et al., 2006). Conventional QSAR models are still limited by high sequence homology within training datasets and insufficient representation of fungal-specific peptides, which may limit predictive generalizability for AFP screening tasks. In this context, improved neural network-based models have been employed to predict AMP activity as AI technologies and AMP dataset quality have improved (Fjell et al., 2009). Predictive models are generally categorized into classification models and regression models (Fang et al., 2024; Yin et al., 2024). Classification models are mainly used to predict antimicrobial activity, whereas regression models can be used to predict variables like minimum inhibitory concentration (MIC), solubility, and stability (Pandi et al., 2023). Machine learning algorithms, including support vector machines (SVM) and random forests (RF), together with peptide physicochemical descriptors, have been widely applied to evaluate candidate peptides (Pandi et al., 2023). For example, machine learning strategies have been used to identify antimicrobial and non-hemolytic β-hairpin cationic peptides (Cbeta-HCPs) (Cruz-Monteagudo et al., 2011). In addition, integrated regression frameworks combining bidirectional long short-term memory (BiLSTM), convolutional neural networks (CNN), and multi-branch models (MBM) have demonstrated improved predictive performance for AMP minimum inhibitory concentration estimation (Chung et al., 2024).

To overcome the limited fungal specificity of generalized AMP prediction systems, Kang and colleagues proposed an AFP-oriented computational prioritization framework integrating one antifungal classification model together with four species-specific activity prediction models targeting major fungal pathogens (Zhang et al., 2022). Importantly, the authors introduced the antifungal index (AFI), a unified scoring system enabling quantitative ranking and systematic prioritization of candidate AFPs across multiple fungal activity models. AFI-guided frameworks demonstrated improved performance compared with CAM, Antifp, and DBAASP for quantitative prediction of antifungal activity and rational lead candidate prioritization. These tools primarily rely on qualitative antimicrobial annotations and activity estimation, which limits their utility for rational candidate prioritization. In this context, the authors developed a multistep computational framework and screened approximately 3.44 million peptide sequences. They established a fungal-specific AFP prioritization framework that integrated quantitative activity prediction and candidate ranking. Collectively, these advances marked a transition from generalized AMP prediction systems toward AFP-specific computational prioritization frameworks.

In 2017, Petra Schneider and colleagues introduced deep learning (DL) frameworks into AMP research through the application of deep neural network architectures for peptide classification and chemical feature analysis (Schneider et al., 2017). Compared with traditional ML approaches, DL-based generative models enable both activity prediction and *de novo* peptide sequence generation (Muller et al., 2018). For example, the Multi-CGAN framework developed by Haoqing Yu et al. was trained using multi-property peptide datasets to generate AMP candidates with optimized antimicrobial features (Yu H. et al., 2024; Yu Q. et al., 2024). Generative models trained on validated peptide datasets may expand accessible peptide sequence space and facilitate the identification of structurally novel antimicrobial candidates (Padi et al., 2025). Nevertheless, AFP-specific validation of generative AI models remains relatively limited because most currently available deep learning frameworks are still predominantly trained on antibacterial peptide datasets rather than fungal-oriented data. Consequently, further development of AFP-specific generative models, fungal activity datasets, and toxicity-aware optimization strategies will likely be required to improve translational predictability and facilitate the development of AFP-oriented generative design frameworks with improved fungal specificity and translational relevance.

### 4.2 Structure-guided optimization of AFPs

Structure modeling and computational simulation techniques provide a critical link between AFP sequence characteristics and their biological activity mechanisms. For fungal target proteins, three-dimensional structure modeling combined with molecular docking can predict the binding patterns and affinities between AFPs and their targets, thereby providing atomic-level evidence for sequence optimization and mechanism-of-action inference. Unlike bacterial membranes, fungal cell envelopes possess multilayer structural architectures enriched with ergosterol, β-glucans, and chitin, which substantially influence AFP binding orientation, membrane penetration, and target accessibility. Consequently, AFP optimization requires simultaneous balancing of fungal selectivity, peptide stability, and biosafety. Recent advances in AlphaFold and RoseTTAFold have substantially improved the accuracy of peptide–protein structural prediction and enabled more reliable interpretation of AFP structure–function relationships, amphiphilic organization, and target-binding interfaces (Baek et al., 2021; Jumper et al., 2021). These structure prediction frameworks further support fungal-specific peptide engineering through residue-level structural interpretation and scaffold re-engineering.

Molecular docking simulates the spatial coordination and energy scoring between peptides or small molecules and targets, revealing potential binding sites and key interacting residues. This method has been widely applied in the screening and design of antifungal candidate molecules. For example, Li et al. performed molecular docking of the antifungal peptide CGA-N12 and its derivative analogs against a three-dimensional model of CtKRE9 (*Candida tropicalis* β-1,6-glucan synthase) (Li G. et al., 2023; Li R. et al., 2023). Based on docking-guided sequence optimization, the authors reduced peptide docking energy and improved antifungal activity against *C. tropicalis*. Several analog candidates exhibited enhanced therapeutic indices and improved antifungal performance. These findings demonstrate that structure-guided interaction analysis may facilitate AFP scaffold modulation through optimization of peptide orientation, residue-level interactions, and fungal target recognition. Collectively, these structure-guided computational approaches represent a central component of interpretable AI-assisted AFP design by linking sequence-level modifications with mechanistic and functional outcomes.

Building upon AFP-specific computational prioritization frameworks, recent studies have increasingly explored structure-guided scaffold re-engineering and domain-level modulation strategies to balance antifungal efficacy with reduced cytotoxicity. In particular, the mesoricin optimization strategy demonstrated that selective attenuation of membrane-disruptive α-helical regions could reduce hemolytic toxicity while preserving antifungal activity mediated by structurally conserved β-sheet-associated functional motifs (Zhang et al., 2024). This research underscored the need for structurally informed and toxicity-aware AFP tuning, and further showed how AFP optimization differs from conventional AMP strategies, which primarily focus on membrane disruption.

Molecular dynamics (MD) simulations also provide insight into the stability and dynamics of AFP–target complexes, as well as conformational changes associated with peptide–target interactions, based on docking results. Interaction stability and binding characteristics of peptide-target complexes can be assessed using parameters such as root mean square deviation (RMSD) values, hydrogen bond stability, and binding free energy under simulated physiological conditions. In AFP studies, MD simulations have been employed to investigate peptide interactions with sterols, particularly ergosterol, in fungal membranes and peptide-induced membrane permeation and remodeling. These membrane remodeling and permeation processes cannot be fully described by static docking results. Several molecular simulation studies have shown preferential interactions of AFPs with ergosterol-rich membrane models, illustrating the role of membrane composition in determining the selectivity of AFPs and their membrane-disruptive activity (Lakshminarayanan et al., 2014). There is also a growing focus on multiscale and coarse-grained MD simulations to examine the diffusion of AFPs across complex fungal cell wall structures rich in β-glucans and chitin (Curto et al., 2021), although experimentally validated AFP-specific studies in this area remain limited.

Despite these advances, currently existing structure-guided AFP optimization frameworks face two major limitations. The first is that most docking, folding, and MD simulations still rely on simplified membrane environments and predominantly static target conformations, which cannot fully capture the dynamic complexities of fungal phospholipid membranes, cell envelopes, and biofilm-associated infection microenvironments. Second, experimentally resolved fungal target structures remain limited for many clinically relevant pathogens, potentially restricting predictive reliability during AFP engineering and validation. Future AFP optimization will require tighter integration of structure prediction, dynamic simulation, and experimental validation.

### 4.3 Activity–toxicity prioritization of AFPs

The assessment of toxicity and pharmacokinetics (ADME / Tox) feasibility and safety are important phases in determining whether an AFP can be clinically translated. The balance between antifungal potency and host toxicity represents one of the major challenges limiting the clinical translation of AFPs. Excessive membrane-disruptive activity may improve antifungal potency; however, it may also increase hemolytic and cytotoxic liabilities. Increasing hydrophobicity, cationic charge density, and amphiphilicity may strengthen interactions with fungal membranes; however, these same physicochemical features may also enhance nonspecific disruption of mammalian cell membranes and increase hemolytic toxicity (Pirtskhalava et al., 2021).

At present, peptide pharmaceuticals may contain threats of hemolytic, cytotoxic, immunogenic, or metabolic instability which might hinder their applicability in the clinic. Scientists have developed different computational safety-screening methods to evaluate peptide candidates and reduce the likelihood of failure during later-stage experimental validation. For example, the ToxinPred2 model developed by Sharma et al. applies machine learning classification strategies based on peptide sequence composition and physicochemical descriptors to evaluate peptide toxicity (Sharma et al., 2022). The model demonstrated high predictive accuracy and Matthews correlation coefficients in independent validation datasets and may support sequence-level toxicity optimization during early-stage peptide screening.

Moreover, the hemolytic capacity of short peptides serves as a key indicator for evaluating peptide safety and is commonly used to estimate the damaging effects of peptide candidates on erythrocyte membranes. Hemolysis-aware prioritization is particularly important for AFPs because membrane-active peptides may damage both fungal and mammalian membranes if selectivity is insufficient. Recent structure-aware models have attempted to improve toxicity prediction by integrating peptide sequence and structural information. For example, ToxGIN uses graph isomorphism networks to incorporate peptide structural representations for toxicity prediction, thereby improving discrimination between toxic and low-toxicity peptide candidates (Yu H. et al., 2024; Yu Q. et al., 2024).

Building upon AFP-specific computational optimization frameworks, recent studies have increasingly explored simultaneous balancing of antifungal activity and hemolytic toxicity. In the mesoricin study, Zhao et al. identified a dual-domain peptide from *Mesorhizobium* sp. using the AFI, then optimized it through α-helix removal and AFI-guided mutations to reduce cytotoxicity while retaining antifungal activity (Zhao et al., 2025). The optimized mesoricin4 variant exhibited potent antifungal activity against various *Cryptococcus* and *Candida* species, with MIC values of 8–16 μg/mL. It also demonstrated enhanced biosafety profiles, reflected by IC50 values above 128 μg/mL against tested human cell lines (Zhao et al., 2025). This case illustrates that AFP-specific computational prioritization can direct peptide design from maximizing antifungal activity alone toward optimizing therapeutic selectivity and toxicity profiles.

In the context of ADMET prediction, many tools were originally developed to predict the ADMET properties of small-molecule drugs, but these tools are increasingly being adapted for peptide therapeutics. However, peptide-based therapeutics have several unique pharmacokinetic limitations, such as susceptibility to enzymatic degradation, limited membrane permeability, rapid systemic clearance, and low bioavailability when administered orally (Klepach et al., 2022). These tools enable prioritization of peptide candidates with improved pharmacokinetic properties and reduced toxicity. These platforms are used to predict peptide absorption, distribution, metabolism, and excretion, as well as assess possible organ toxicity risks. These *in silico* ADMET analyses support early prioritization of AFP candidates with improved safety and developability.

### 4.4 Target-aware interaction modeling of AFPs

Target-aware computational interaction modeling has become increasingly important for mechanistic interpretation of AFP activity and fungal selectivity. Unlike conventional antibacterial peptide systems, AFP–fungal target interactions are additionally influenced by fungal cell wall architecture, ergosterol-rich membrane composition, and biofilm-associated diffusion barriers (Rodrigues, 2018). Consequently, modern interaction modeling strategies increasingly integrate molecular docking, molecular dynamics (MD) simulation, and membrane interaction modeling to characterize AFP–target recognition and mechanistic behavior at the atomic level (Hospital et al., 2015).

Molecular docking is widely used to predict binding conformations, interaction interfaces, and energy profiles between AFPs and fungal targets. By estimating binding affinity and identifying key interacting residues, docking analysis may facilitate mechanistic interpretation and rational optimization of AFP candidates. In recent years, docking-guided AFP optimization has increasingly focused on fungal membrane-associated proteins and cell wall biosynthesis targets involved in antifungal resistance and pathogenicity (Pagadala et al., 2017). A representative example was reported by Li et al., who investigated the interaction between the antifungal peptide CGA-N12 and CtKRE9, a β-1,6-glucan synthesis-associated protein from *Candida tropicalis* (Li G. et al., 2023; Li R. et al., 2023). Through molecular docking analysis, the authors identified key peptide–target interaction residues and optimized peptide analogs by reducing docking energy and improving target-binding stability. Peptide variants with design modifications showed increased antifungal effects and better therapeutic indices against *C. tropicalis*, showing the potential for docking-guided AFP optimization against fungal targets.

In addition to static docking analysis, molecular dynamics simulations can help clarify the dynamics of conformational stability and their interactions within membranes, as well as the behavior of peptides when modified under physiological conditions. Root means square deviation (RMSD), the stability of hydrogen bonds, solvent accessibility, and t binding free energy are used to assess the stability of AFP-target interactions over time (Hospital et al., 2015). MD simulations have been used to analyze peptide interactions with ergosterol-containing fungal membranes and study antifungal selectivity-related membrane remodeling in AFP-related systems (Lakshminarayanan et al., 2014). By performing these simulations provide improved understanding of peptide orientation and membrane interactions and how they orient and disrupt membrane barriers, as well as how they selectively breach the barriers of a fungus. Other studies also address the role of foam peptide (FP) in the interactions and permeation of the fungus membrane. For example, Wang and coworkers showed how some of the fungal peptides were able to give a fungistatic effect to the well-known pathogen *Candida albicans* due to facilitated interaction and integration with ergosterol-rich lipid rafts and foam, in conjunction with some the multi-omics of membranes and chemical barriers which also helped to support the findings (Wang S. et al., 2025; Wang Y. et al., 2025). These results highlight the significance of modeling membrane interactions to interpret the mechanisms of AFP selectivity and fungal membrane disruption.

Additionally, the modeling of membrane interactions has become increasingly important for the mechanistic understanding of AFP selectivity, particularly owing to differences in the lipid composition of fungal and mammalian membranes. According to the interfacial activity model for membrane-active peptides, peptide function is highly dependent on the ability of peptides to partition into membranes and interact with membrane surfaces, rather than isolated binding events involving membrane receptors (Wimley, 2010). These concepts are particularly relevant to AFP systems, as fungal membrane ergosterol composition and diffusion barriers may impede peptide penetration and influence antifungal efficacy. In recent reviews, it has been noted that AFP activity involves a combination of membrane disruption, intracellular targeting, oxidative stress induction, and interference with biofilm formation, as opposed to the inhibition of a single pathway (Gong et al., 2024).

Collectively, target-aware interaction modeling frameworks provide increasingly interpretable strategies for mechanistic elucidation and rational refinement of AFP candidates.

### 4.5 Mechanism-guided iterative refinement of AFPs

Mechanism-guided iterative refinement has emerged as an increasingly important strategy for improving the antifungal efficacy, biosafety, and translational developability of AFP candidates (Das et al., 2021; Mookherjee et al., 2020). Unlike early-stage AFP discovery workflows that primarily focused on identifying peptides with antifungal activity, modern AFP engineering increasingly emphasizes iterative optimization through continuous integration of activity prediction, structural interpretation, toxicity prioritization, and mechanistic feedback. Consequently, AFP optimization has gradually evolved from single-parameter screening toward multi-objective computational refinement frameworks balancing antifungal potency, selectivity, structural stability, pharmacokinetic properties, and biosafety (Porto et al., 2018).

Recent advances in artificial intelligence and computational peptide engineering have substantially accelerated iterative AFP optimization (Das et al., 2021). Multi-objective optimization incorporates antifungal activity, hemolytic toxicity, membrane selectivity, and peptide stability during AFP redesign (Porto et al., 2018). This is particularly pertinent for AFP systems because enhanced fungal membrane disruption may result in increased cellular toxicity toward mammalian membranes, while also reducing peptide stability under physiological conditions (Mahlapuu et al., 2016). Therefore, AFP-oriented refinement increasingly relies on closed-loop computational optimization that integrates sequence redesign, structural evaluation, activity prediction, toxicity screening, and experimental feedback into iterative engineering cycles (Das et al., 2021). An example of mechanism-guided AFP refinement is found in the mesoricin optimization framework reported by Zhao et al. (2025). The authors used a combination of antifungal index (AFI)-guided screening, structural redesign, toxicity-aware prioritization, and experimental testing to optimize mesoricin-derived peptides. The first-generation peptide mesoricin1 exhibited strong membrane-disruptive activity, but also high cytotoxicity and significant hemolytic liability. The authors used a gradual redesign approach to reduce the α-helical membrane-disruptive region to enhance biosafety. In addition, AFI-guided mutations were used to restore both antifungal activity and membrane permeability (Zhao et al., 2025). The optimized variant mesoricin4 retained potent activity against multiple *Candida* and *Cryptococcus* species while substantially reducing mammalian cytotoxicity and hemolysis. This process demonstrates how AFP engineering can utilize mechanism-aware computational refinement to optimize the balance between antifungal activity, therapeutic selectivity, and developability.

Iterative AFP optimization is increasingly incorporating mechanistic and membrane-associated simulations. Feedback from these simulations, such as the structural interpretation of target interactions, membrane permeabilization behavior, and ergosterol selectivity, will likely influence subsequent sequence redesign and functional optimization. AI-assisted sequence generation combined with mechanistic filtering and experimental validation is expected to accelerate AFP optimization and may reduce reliance on for empirical trial-and-error screening during AFP development (Das et al., 2021). These closed-loop refinement strategies suggest that AFP development is becoming increasingly adaptive, moving beyond static predictive workflows toward adaptive computational-experimental engineering systems.

Modern frameworks for the refinement of AFPs include the assessment of antifungal potency along with key developability parameters. These parameters include peptide solubility, proteolytic stability, tendency to aggregate, membrane permeability, and pharmacokinetic behavior (Klepach et al., 2022). This transition reflects a broader shift in peptide engineering from activity-centered optimization toward translationally informed therapeutic design (Mookherjee et al., 2020). Thus, mechanism-guided AFP optimization not only enhances antifungal potency but also enables the selection of peptide candidates with improved translational potential.

Mechanism-based design refinement has become an important component of contemporary antifungal peptide (AFP) engineering. It combines artificial intelligence (AI)-based sequence redesign, structural and mechanistic analyses, toxicity-aware prioritization, membrane interaction modeling, and rigorous experimental validation. This integration helps support rational AFP design and translational development.

### 4.6 Current limitations and future perspectives

Despite advances in AI-powered peptide engineering, AFP development continues to face several important challenges. These challenges arise from limited fungal-specific datasets, a lack of mechanistic interpretability, and a persistent gap between computational prediction and translational validation. The discovery of AFPs has benefited substantially from recent advances in computational frameworks. Unfortunately, AFP prediction models are often adapted from antibacterial AMP datasets rather than trained on large fungal-specific datasets. Research on antibacterial peptides has generated large and diverse datasets, while AFP-related datasets remain comparatively limited, particularly with respect to quantitative antifungal activity, fungal subtype specificity, and *in vivo* pharmacokinetic and toxicity studies. These limitations may reduce model generalizability and, in turn, can affect the predictive performance of AFP design frameworks and computational screening systems (Mookherjee et al., 2020).

Another major limitation is the lack of experimentally resolved fungal target structures. Recent improvements in AlphaFold and cryo-electron microscopy have substantially advanced structural biology. Nevertheless, high-resolution structural information is not yet available for many fungal membrane proteins, biofilm-associated targets, and cell wall synthesis complexes. As a result, important limitations remain regarding docking precision, the interpretation and accuracy of molecular interactions, and the reliability of mechanistic modeling for many AFP–fungal systems (Jumper et al., 2021). Furthermore, contemporary molecular dynamics simulations tend to rely on simplified membrane models. Consequently, accurately modeling ergosterol heterogeneity, lipid microdomains, and diffusion barriers associated with the cell wall remains important for understanding the physicochemical complexity of fungal membranes (Rodrigues, 2018).

Improvements in computational prediction models may facilitate AFP optimization; however, successful translation ultimately requires experimental and clinical validation. Translational AI models increasingly incorporate antifungal activity, docking affinity and interaction dynamics with target proteins, and toxicity prediction. Clinically relevant AFP candidates are computationally predicted to exhibit favorable pharmacokinetics, proteolytic stability, immunocompatibility, formulation feasibility, and scalability for industrial production. This highlights the persistent gap between computational drug design and real-world therapeutic developability (Klepach et al., 2022). In addition, the AFP evaluation criteria remain insufficiently standardized in the literature. Significant inconsistencies remain, including differences in datasets, activity thresholds, selection of fungal species, toxicity tests, and benchmarking, that make it difficult to assess reproducibility and limit cross-platform comparability.

Future AFP development will demand better integration of machine learning, artificial intelligence, structural biology, mechanistic modeling, and experimental validation to create more effective and selective antifungal therapeutics (Das et al., 2021). Improvements in fungal structural biology and high-throughput experimental systems may allow for the rational optimization of AFP candidates that can be advanced toward clinical translation. Importantly, predictive improvements will contribute substantially to AFP development, but advances in mechanistic understanding of biological systems, together with translational validation, will be essential for progress. The integration of mechanistic insight and closed-loop computational design may drive a paradigm shift in the development of rational and clinically applicable AFPs, moving beyond empirical peptide screening approaches.

## 5 *In vitro* and *in vivo* experimental evidence

AFPs display multiple antifungal mechanisms of action, which may reduce the likelihood of antifungal drug resistance development in multidrug-resistant fungi (MDR) (Buda De Cesare et al., 2020; Mahlapuu et al., 2016). In addition to causing membrane damage, AFPs can disrupt intracellular metabolic homeostasis, induce oxidative stress and interfere with intracellular signaling pathways. Consequently, AFPs may achieve antifungal efficacy at lower concentrations than many conventional antifungal agents (Freitas and Felipe, 2023; Lupetti et al., 2008). Recent advances in AI-assisted screening, structural optimization, and multimodal experimental validation have accelerated AFP development against clinically important fungal pathogens, including *Candida albicans*, *Candida auris*, and *Aspergillus* spp. (Lee et al., 2022; Sun et al., 2024). Experimental validation of AFPs, as detailed in Figure 2, relies on four elements: (i) *in vitro* evaluation of MIC, biofilm inhibition, hemolysis, and cytotoxicity; (ii) assessment of cell wall and membrane disruption, as well as target-site interactions; (iii) *G. mellonella* and mammalian models for assessing fungal burden, host survival, toxicity, and wound healing; and (iv) integrated computational and experimental analyses for systematic evaluation of AFP antifungal efficacy, safety, and translational potential.

### 5.1 Activity against *Candida* spp. (including *Candida auris* and azole-resistant strains)

*Candida* species are among the most common fungal pathogens responsible for invasive candidiasis, particularly in immunocompromised individuals. The conventional antifungal agents, such as azoles and echinocandins, are widely used clinically, and resistance to these agents is increasing. Therefore, alternative antifungal agents, such as AFPs, are being explored for the treatment of resistant *Candida* infections (Freitas and Felipe, 2023). Several studies have confirmed that AFPs exhibit *in vitro* antifungal activity against *Candida albicans* and other *Candida* species, including resistant strains. VLL-28 was designed from an archaeal protein sequence and demonstrated antifungal activity against 10 clinically isolated *Candida* spp. VLL-28 also inhibits biofilm formation in *C. albicans* and *C. parapsilosis* (Roscetto et al., 2018). VLL-28 binds to the surfaces of fungal cells and disrupts fungal cell wall and membrane integrity. Lee et al. developed another synthetic peptide, PS1-2, which also demonstrated potent activity against fluconazole-resistant *C. albicans*, with MIC values of 16–32 μM. PS1-2 exhibited antifungal and anti-inflammatory activity without detectable resistance induction (Lee et al., 2022). The positive charge, amphiphilic structure, and other physicochemical properties of AFPs contribute to their antifungal activity because they facilitate destabilization and permeabilization of fungal membranes. Recent evidence demonstrating the ability of the synthetic peptide C14R to disrupt membranes of *C. auris* clinical isolates is compelling. Low MIC values in the μg/mL range were reported for C14R against clinical isolates of *C. albicans* and *C. auris* (Velez et al., 2024). Additionally, C14R effectively compromises fungal membranes, inhibits biofilm formation, and exhibits synergistic effects with azoles, thereby enhancing sensitivity of drug-resistant strains to antifungal therapies (Velez et al., 2024). These antifungal mechanisms include oxidative stress induction, disruption of intracellular metabolism, and membrane destabilization.

Formation of *Candida* biofilms contributes to antifungal resistance and persistence of chronic infections. Limited penetration of traditional antifungal agents into fungal biofilms enables biofilm persistence. Due to their amphiphilic properties and relatively small size, AFPs may penetrate and disrupt fungal biofilm matrices. Roscetto et al. reported that VLL-28 not only targets planktonic fungal cells but also exhibits inhibitory activity against biofilm formation in clinically isolated *C. albicans* and *C. parapsilosis* strains, suggesting that its anti-biofilm activity may involve disruption of intercellular adhesion and biofilm matrix integrity (Roscetto et al., 2018). In a study of fluconazole-resistant *C. albicans* by Lee et al., PS1-2 demonstrated inhibitory effects against dense extracellular matrix-associated biofilms. On the molecular level, PS1-2 also induced cellular stress responses, further illustrating that AFPs exert antifungal activity through multiple mechanisms to inhibit *Candida* biofilms (Lee et al., 2022). Investigation of AFPs has become increasingly important because *Candida auris* has emerged as a multidrug-resistant fungal pathogen. While maintaining activity against highly resistant strains (with no significant correlation observed between MIC values and fluconazole resistance), the C14R peptide reported by Vélez et al. demonstrated fungicidal activity against 99 *C. albicans* and 105 *C. auris* clinical isolates (Velez et al., 2024). These findings suggest that the mechanism of action of C14R diverges from the conventional ergosterol-targeting resistance pathways by preferentially disrupting fungal membranes rather than inhibiting ergosterol biosynthesis. Importantly, the study also showed that combination treatment with C14R and fluconazole reduced MIC values for both agents (FIC ≤ 0.5) (Velez et al., 2024). These findings further suggest that anti-biofilm and fungicidal activities may act synergistically, indicating that combining AFPs with conventional antifungal agents may improve treatment efficacy against resistant fungal pathogens while reducing dosage requirements and minimizing treatment-associated toxicity.

Collectively, current studies demonstrate that diverse AFPs exhibit antifungal activity against both susceptible and drug-resistant *Candida* species. AFPs commonly inhibit biofilm formation and disrupt fungal membrane integrity through membrane-associated disruption and intracellular targeting mechanisms. Several AFPs have also demonstrated synergistic activity with multiple classes of antifungal agents, supporting their potential application in the treatment of resistant *Candida* infections as combined therapy. Continued interest remains in the investigation of AFPs for the treatment of *C. auris* and other multidrug-resistant fungal pathogens.

### 5.2 Activity against *aspergillus* species and other molds

Invasive aspergillosis caused by *Aspergillus fumigatus* is associated with high morbidity and mortality in immunocompromised patients. Increasing resistance to conventional antifungal agents highlights the need for novel therapeutic strategies. The ability of lipopeptide and peptide antibiotics to interact with and disrupt fungal membranes, and induce stress responses, provides multiple mechanisms that may overcome fungal persistence and antifungal resistance, particularly in *A. fumigatus* and other filamentous fungi. AFP research has primarily focused on yeast-like fungi, especially *Candida* species. Although studies on filamentous fungi remain limited, evidence suggests that some host-derived and synthetic peptides exhibit inhibitory activity against *Aspergillus* species. Lupetti et al. evaluated the *in vitro* antifungal activity of several AFPs derived from human lactoferrin, histones, and ubiquicidin (UBI). These peptides exhibited inhibitory activity against both spores and hyphae of *A. fumigatus* in a dose-dependent manner. Some of these peptides demonstrated particularly strong activity against fungal spores, including hLF(1–11), dhvar5, and UBI 18–35 (Lupetti et al., 2008). These findings demonstrated that host defense peptides not only suppress *Candida* species but also directly kill filamentous fungi including *A. fumigatus*. More recently, two synthetic short-chain antimicrobial lipopeptides (C14-NleRR-NH₂ and C14-WRR-NH₂) were reported to exhibit *in vitro* antifungal activity against azole-resistant *A. fumigatus* strains (Fioriti et al., 2022). Experimentally, these synthetic lipopeptides exhibited MIC values ranging from 8 to 16 μg/mL. A significant dose-dependent inhibition was observed in both time-kill assays and XTT metabolic activity assays (Fioriti et al., 2022). Optical microscopy further confirmed marked inhibition of hyphal growth in treated resistant strains. Overall, these studies suggest that AFPs may serve as a novel therapeutic strategy for resistant *Aspergillus* infections.

Some host innate immune peptides may also modulate host immune responses and contribute to host defense against invasive fungal infections. LL-37 (Table 1) exhibits context-dependent activity against *A. fumigatus*. In certain experimental settings, LL-37 demonstrated direct antifungal activity against *A. fumigatus* while also modulating inflammatory responses. Conversely, under different experimental conditions, LL-37 was associated with enhanced fungal invasion (Luo et al., 2019). These inconsistent findings suggest that the antifungal properties of immunomodulatory peptides may depend on environmental conditions as well as the fungal morphogenetic stage (spores versus mycelium) (Luo et al., 2019). AFPs have also demonstrated inhibitory activity against additional non-*Aspergillus* fungal genera. Other researchers have noted that AFPs exhibit broad-spectrum antifungal activity against most molds and filamentous fungi (Buda De Cesare et al., 2020). These peptides may disrupt fungal membranes, induce pore formation, and elicit stress responses (Buda De Cesare et al., 2020). Despite the limited number of systematic *in vitro* studies for non-*Aspergillus* filamentous fungi, available studies suggest that AFPs exhibit measurable *in vitro* antifungal activity for a number of fungal species. Recently conducted experimental trials continue to support the antifungal activity of AFPs for *Aspergillus* and other mold species. Additionally, both naturally occurring and synthetic or engineered peptides exhibit *in vitro* antifungal activity against *A. fumigatus*, especially the resistant strains. Although only a limited number of studies have focused on the *in vivo* efficacy and mechanism of action, the *in vitro* studies offer preliminary evidence supporting potential therapeutic application against invasive fungal infections.

### 5.3 Synergistic effects with existing antifungal agents

ToAP2 and fluconazole together showed a cooperative inhibition of *C. albicans* biofilms. A checkerboard microdilution assay was used to investigate the interaction between ToAP2 and fluconazole. The combination of these two agents significantly inhibited *C. albicans* biofilm formation compared with either agent alone. These findings indicate synergistic interactions (FICI < 0.5) (do Nascimento Dias et al., 2024). These findings further suggest that AFPs may improve antifungal activity by facilitating antifungal penetration, as well as increasing membrane permeability through membrane disruption and biofilm matrix destabilization. In addition, studies involving AMP-17, an antifungal peptide derived from housefly larvae, showed that combination treatment with fluconazole increased the susceptibility of biofilm-forming *C. albicans*. According to checkerboard analyses, in most of the tested clinical isolates, a greater biofilm inhibition was observed with the combination of AMP-17 and fluconazole compared with either agent alone (Sun et al., 2024). Furthermore, the combination notably reduced the effective therapeutic concentrations required, indicating that AFPs may serve as promising adjunctive therapies to azole-based antifungal agents for delaying the development of antifungal resistance (Sun et al., 2024). These findings are consistent with previous studies demonstrating that combining AFPs with azole antifungal agents may improve biofilm inhibition and enhance antifungal susceptibility in resistant strains (Rodriguez-Castano et al., 2023).

Synthetic defensin-derived peptides and γ-core motif peptides have demonstrated synergistic activity with amphotericin B (Souza et al., 2024). Previous studies further demonstrated that peptide fragments derived from potato defensin PvD₁ (e.g., γ₃₁–₄₅PvD₁^++^ and γ₃₃–₄₁PvD₁^++^) exhibited enhanced *in vitro* antifungal efficacy when combined with amphotericin B in checkerboard assays (Souza et al., 2024). The observed synergistic effects between AFPs and polyene antifungal agents may result from the ability of AFPs to disrupt fungal membranes, increase antifungal agent permeability, and enhance antifungal efficacy against resistant fungal cells and biofilms. Bezerra et al. further reported that the semi-synthetic AMPs Mo-CBP₃-PepI and Mo-CBP₃-PepIII significantly enhanced antibiofilm activity against *C. albicans* and *C. parapsilosis* when combined with conventional antifungal agents such as nystatin and itraconazole (Bezerra et al., 2022). The study demonstrated that peptide–drug combinations were significantly more effective than single-agent treatment in preventing and disrupting biofilm formation. Specifically, biofilm inhibition ranged from 30 to 45% with single agents and 79–98% with combination treatment, depending on the specific peptide–drug combination (Bezerra et al., 2022). These additional findings indicate that AFPs may act synergistically with conventional antifungal agents, particularly in the treatment of biofilm-associated fungal infections. AFPs, together with conventional antifungal agents, may augment drug permeability, destabilize biofilms, and provide complementary antifungal mechanisms. More importantly, combinations of AFPs with conventional antifungal agents may exhibit enhanced antifungal efficacy compared with individual agents alone and may help delay the development of antifungal resistance.

### 5.4 *In vivo* model validation

*In vivo* validation is essential for evaluating the translational potential of AFPs. Commonly used *in vivo* models include the invertebrate *Galleria mellonella* (*G. mellonella*) infection model and mammalian models of systemic fungal infection, particularly murine models. These models are widely used to evaluate the antifungal efficacy, host immune responses, toxicity, and pharmacodynamic properties of AFPs.

*Galleria mellonella* has emerged as an important surrogate *in vivo* model for evaluating fungal infections and antifungal therapies because of its low cost, ease of handling, ability to survive at 37 °C, and innate immune responses that partially resemble those of vertebrates. This model has been widely applied for *in vivo* efficacy evaluation against both yeast-like fungi, including *Candida* and *Cryptococcus* species, and filamentous fungi such as *Aspergillus* species (Jemel et al., 2020). *In silico*-designed AFPs such as ISDPs have also demonstrated efficacy in *G. mellonella* infection models. These designed peptides not only exhibited potent *in vitro* anti-*Candida* activity but also significantly improved wax moth survival following infection without apparent toxicity, supporting their translational potential for further preclinical development (Ciociola et al., 2021). The Jelleine-II derivative peptide JII-R1 was also reported to improve survival in a *G. mellonella Candida* infection model (Perez-Rodriguez et al., 2025). JII-R1 demonstrated both antifungal efficacy and low toxicity against multiple *Candida* species *in vivo*. Several systematic reviews have highlighted the advantages of *G. mellonella* as an *in vivo* model for evaluating antifungal agents (peptides, combined treatments), thereby supporting its utility for preliminary *in vivo* AFP screening (Giammarino et al., 2024).

Although *G. mellonella* is a useful model for rapid screening of candidate peptides, its immune and metabolic systems differ substantially from those of mammals. Nevertheless, mammalian models, and specifically murine systemic models of *Candida* infection, remain essential for evaluating the *in vivo* effectiveness, safety, and potential immunomodulatory impact of AFPs. The peptide AMP-17, from *Musca domestica*, significantly improved host survival in a model of *C. albicans* invasive candidiasis, reduced renal fungal burden by approximately 90%, and modulated post-infection inflammatory responses. These findings highlight the multifaceted therapeutic effects of AMP-17. The study also employed an invasive pulmonary aspergillosis (IPA) model in order to address the therapeutic activity of AMP-17, alone and in combination with fluconazole (Sun et al., 2024). Host defense peptide mimetics also demonstrated potent *in vivo* antifungal activity, reducing renal fungal burden by up to 3–4 log₁₀ CFU/g in CD-1 mice with invasive candidiasis, supporting the therapeutic potential of peptide-based antifungal agents (Chowdhury et al., 2018). In murine models of drug-resistant *C. albicans* infection, the synthetic peptide AKK8 demonstrated significant therapeutic potential, highlighting the importance of amino acid-based peptide optimization as an approach to enhance *in vivo* antifungal activity (Wubulikasimu et al., 2020). In a mouse model of oral candidiasis, the halocidin-derived HG1 peptide significantly reduced the fungal burden, suggesting that AFPs may be effective against both systemic and localized fungal infections (e.g., *mucosal candidiasis*) (Shin et al., 2013).

Collectively, *in vivo* studies demonstrate that AFPs exhibit potent antifungal activity in experimental infection models by reducing fungal burden and improving host survival. These findings support further preclinical and translational development of AFP-based antifungal therapeutics. Murine models additionally enable simultaneous evaluation of antifungal efficacy, toxicity, pharmacokinetics, and immunomodulatory effects. Although *G. mellonella* is widely used as a high-throughput *in vivo* screening model, its immune and metabolic systems remain less complex than those of mammals, thereby requiring validation in mammalian models before clinical translation can be considered. Nevertheless, systematic *in vivo* evaluation against multidrug-resistant fungal pathogens remains limited in the current literature, highlighting an important direction for future AFP research.

## 6 Translational and clinical challenges

**Figure 3:**
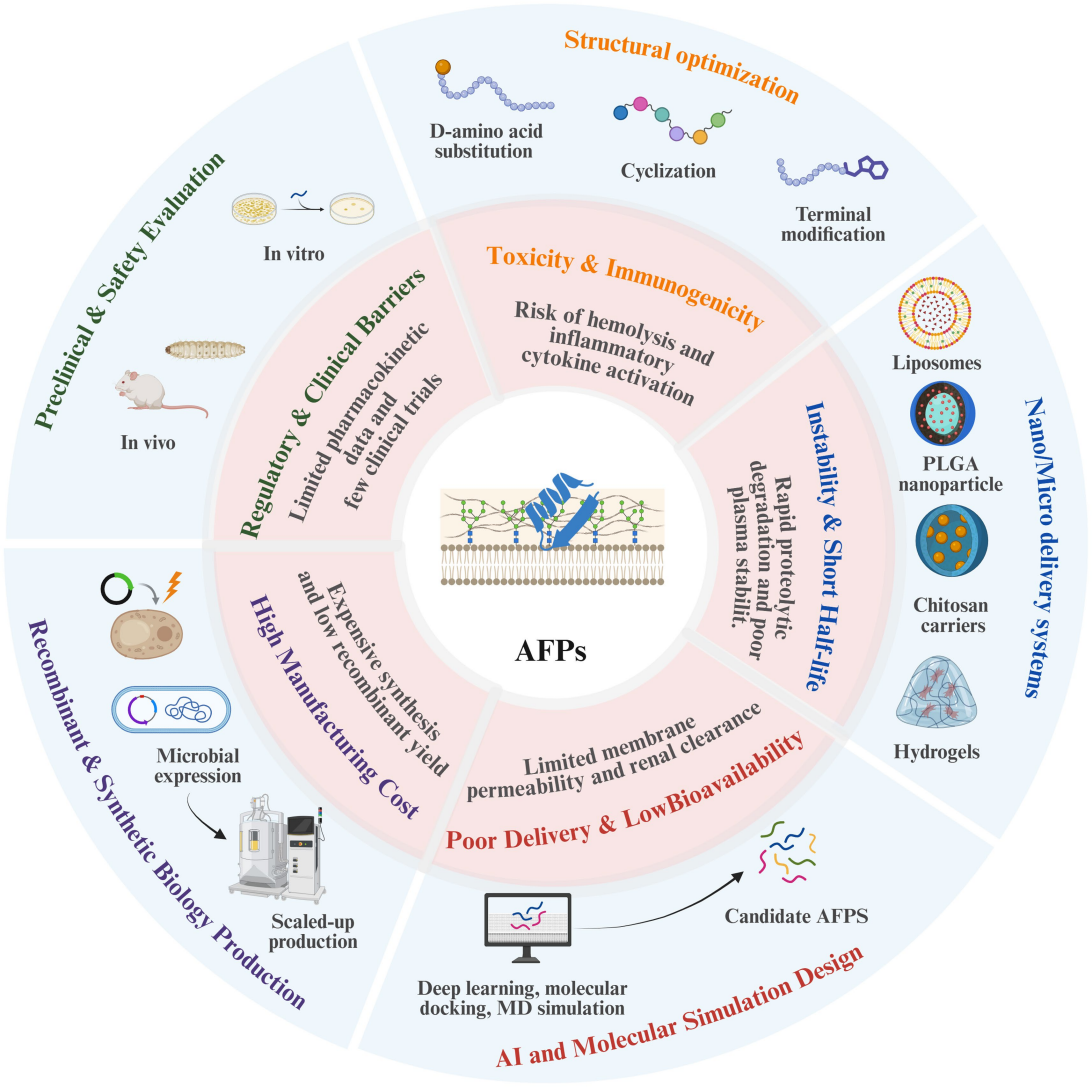
Integrated translational roadmap of natural AFPs against multidrug-resistant fungal pathogens. Major translational barriers and engineering strategies in antifungal peptide (AFP) development against multidrug-resistant fungal pathogens. Key limitations include toxicity and immunogenicity, proteolytic instability, short plasma half-life, limited membrane permeability, poor bioavailability, manufacturing cost, and insufficient pharmacokinetic and clinical data. Engineering approaches include structural optimization (D-amino acid substitution, cyclization, terminal modification), nano- and micro-delivery systems (liposomes, PLGA nanoparticles, chitosan carriers, hydrogels), AI-assisted molecular design, scalable recombinant production, and preclinical safety evaluation *in vitro* and *in vivo*.

Although AFPs show considerable potential for antifungal therapy, only a limited number have advanced to clinical evaluation. Major translational challenges include drug safety, stability, delivery strategies, manufacturing cost, and regulatory considerations (As demonstrated by Figure 3) (Sharma et al., 2025). An example is HLF (1–11) which is a peptide designed to attack fungal infections in seriously immunocompromised individuals, but the development of more in-depth research in this field has stalled (Sierra et al., 2017). A similar case is CZEN002 that went into Phase II clinical trials mainly to address vulvovaginal candidiasis (Fernandez de Ullivarri et al., 2020). No recent clinical development updates regarding this compound have been reported.

### 6.1 Toxicity and pharmacokinetic challenges

Most of the potential AFPs available today are toxic to human organs or blood. Because mammalian and fungal cells share conserved eukaryotic cellular features, it remains challenging to develop AFPs that selectively target fungal cells without affecting mammalian cells (Cresti et al., 2024). Conventional antifungal agents commonly administered to patients with invasive fungal infections, including azoles and polyenes, are associated with nephrotoxicity (Tverdek et al., 2016). An extract of *Bacillus subtilis* has also been demonstrated to be highly hematotoxic in experiments (Aranda et al., 2005). Fungal cell wall-targeting antifungal agents generally exhibit favorable safety profiles because fungal cell wall components are absent in mammalian cells. For example, caspofungin, an echinocandin antifungal semisynthetically derived from pneumocandin B₀ produced by Glarea lozoyensis, selectively inhibits β-(1,3)-D-glucan synthesis and exhibits relatively low host toxicity (Szymanski et al., 2022). Moreover, nucleic acid-targeting compounds such as actinomycin D may induce DNA damage (Hamill, 2013).

In addition, a major barrier to AFP clinical translation is their unfavorable pharmacokinetic profile, due to rapid proteolytic degradation *in vivo*, as well as short circulating half-lives and rapid systemic clearance. Examples of transmembrane AFPs are P7 (based on ppTG20), which penetrate fungal cells and enter into the cell nucleus and bind DNA, resulting in genomic inhibition and structural damage (Li et al., 2016). Blapstin has been reported to inhibit both *C. albicans* and *Trichophyton rubrum* at MIC 7 μM and 5.3 μM respectively, which was isolated from the medicinal beetle *Blaps rhynchopetera* (Zhang et al., 2023). Nevertheless, it is highly susceptible to degradation by plasma proteases *in vivo* and has pharmacokinetic issues relating to low oral bioavailability and stability. Table 1 highlights that spider-derived Gomesin also contains highly cationic physicochemical properties. Although it exhibits antifungal activity, it may also interact with host cell membranes, producing hemolytic and cytotoxic effects (Bolosov et al., 2024). Its derivative DsGom is less toxic, however, additional structural changes are needed to minimize its toxicity further (Bolosov et al., 2024). Therefore, reducing AFP-associated toxicity remains a major research priority in this area. Methods used involve chemical alteration of AFPs or creation of drug delivery systems.

Based on the structural properties of AFPs, they may be modified and optimized in order to increase the antimicrobial activity, improve serum stability, and decrease the toxicity. To begin with, biological activity can be increased by the addition, deletion, or replacement of amino acid residues within the AFP sequence. Nonetheless, balancing antifungal activity, stability, and toxicity is critical, since it is possible to lose activity when attempting to increase stability (Li G. et al., 2023; Li R. et al., 2023). As an example, WRK-30, which is a derivative of the CLEC3A sequence, has very low cytotoxicity to eukaryotic cells (Meinberger et al., 2023). Following the introduction of a sulfonyl-γ-amino acid structural unit into Feleucin-K3, CF3-K11 exhibits markedly enhanced antimicrobial activity and serum stability while achieving 8–9-fold enhanced serum stability and reduced susceptibility to resistance development (Guo et al., 2024). Other researchers replaced L-amino acids with D-amino acids in AMPs, significantly enhancing AFPs’ stability against proteases. Chen et al. substituted L-arginine with D-arginine in HBcARD, markedly reducing its hemolytic toxicity (Chen et al., 2016). Jlenia Brunetti et al. also found that this approach resulted in modified AFPs exhibiting both bactericidal and anti-inflammatory activities, along with enhanced resistance to proteolytic degradation (Brunetti et al., 2020). However, excessive modification, substitution, or inappropriate modification of amino acids may lead to cytotoxicity and increased immunogenicity in AMPs. *In vivo* experiments with DP06 (a derivative of the cationic AMP Pep05) synthesized by Lu et al. using this method showed substantially reduced antifungal activity while increasing toxicity (Lu et al., 2020). The formation of cyclic structures through disulfide bonds or lactones, known as cyclization, enhances the protease stability and selectivity of AFPs while increasing their cell membrane permeability (Riahifard et al., 2018; Schneider et al., 2014). For instance, researchers designed an amphiphilic, enantiomeric L-cyclic peptide that exhibits no cytotoxicity and exhibits efficient cell penetration with minimal cytotoxicity (Mandal et al., 2011). Other modification methods include glycosylation, PEGylation, and esterification (Bellavita et al., 2023). Modifying AFPs presents a complex challenge requiring a delicate balance among stability, antimicrobial activity, hemolytic toxicity, and production costs. AI-assisted optimization strategies may facilitate AFP structural refinement to address these limitations. For instance, Dong-in Kim et al. designed a monomeric pseudo-isolated α-helix (mPIH) system, which may provide a promising platform for the development of stable α-helical peptide therapeutics (Kim et al., 2022).

### 6.2 Delivery systems and formulation strategies

AFPs exhibit broad-spectrum antifungal activity and relatively low resistance potential, but their clinical translation faces a series of pharmacokinetic and delivery challenges. Overall, drug delivery strategies show greater potential than structural modifications in overcoming issues such as *in vivo* toxicity, immunogenicity, and stability deficiencies of AFPs (Jallouk et al., 2015). Nano/micron-scale delivery systems, including liposomes, polylactic acid-hydroxyacetic acid copolymer (PLGA) nanoparticles, chitosan carriers, and hydrogels (Figure 3), are employed to enhance the *in vivo* stability, bioavailability, and pharmacokinetic properties of AFP-based agents.

Liposome-based systems represent the first FDA-approved clinical application of nanomedicine delivery platforms. These systems enhance drug solubility, protect the peptides against enzymatic degradation, enable targeted delivery and controlled release, thereby improving therapeutic efficacy under complex infection conditions (Zheng et al., 2025). AmBisome^®^ (liposomal amphotericin B) was the first FDA-approved liposomal antifungal formulation. Encapsulation of amphotericin B within lipid vesicles improves drug solubility and bioavailability while significantly reducing nephrotoxicity compared with conventional amphotericin B formulations (Ellis, 2002). Similarly, liposomal formulations of voriconazole significantly improve its solubility in aqueous solution and tissue distribution thereby improving antifungal efficacy while reducing toxicity (Veloso et al., 2018). Studies show that liposomes containing Histatin 5 protect peptides from proteolytic degradation by salivary proteases but retain their antifungal activity against *Candida* over 72 h (Zambom et al., 2019). Recently, surface-functionalized liposomes have emerged as a promising strategy for targeted AFP delivery with fungal cell wall-specific ligands to precisely deliver AFPs to treat multidrug-resistant fungal infections. Dectin-1, a C-type lectin receptor that specifically binds to fungal cell wall β-glucan, was used to modify AmBisome liposomes by adding a β-glucan binding domain to the liposome surface (Ambati et al., 2019). This modification significantly improved binding capacity and antifungal effects on both *Aspergillus fumigatus* and *Candida albicans* compared to unmodified liposomes. Based on this, Meagher et al. extended the concept of the DectiSomes: using C-type lectin receptors (e.g., Dectin-1 or Dectin-2) as broad-spectrum fungal recognition ligands and directing them to the fungal cell surface containing conserved polysaccharide structures (Meagher et al., 2023). This strategy increases drug concentration at the site of action and reduces nonspecific interactions with mammalian cells, hence improving therapeutic index and reducing systemic toxicity. Nevertheless, conventional liposomes are still associated with the risk of membrane instability and premature drug leakage in biological fluids. In recent years, controlled antifungal drug and peptide release kinetics may be achieved through outer polymer coating and the construction of pathogen-responsive composite systems, thereby improving *in vivo* stability and targeting efficiency. For instance, Sulastri et al. employed a chitosan/alginate layer-by-layer (LBL) self-assembly technique to double-layer coat nystatin liposomes, resulting in increased encapsulation efficiency and enhanced antifungal activity against *Candida albicans* and improved storage stability (Sulastri et al., 2025). Furthermore, Vera-González et al. developed a responsive hydrogel degradable by fungal-secreted aspartic proteases, with embedded liposomes to achieve infection microenvironment-triggered release. This system undergoes scaffold degradation in the presence of fungal-secreted enzymes, subsequently releasing the antifungal drug loaded within the liposomes. It effectively reduced fungal burden *in vitro* while exhibiting good biocompatibility with mammalian cells (Vera-Gonzalez et al., 2024).

Compared to systemic delivery platforms such as liposomes, hydrogels are more suitable for local infection settings, particularly in oral mucosa, wounds, and biofilm-associated infections. The major challenges with AFPs are short *in vivo* half-lives, protease sensitivity, and limited local retention time. Hydrogels provide a protective microenvironment for AFPs through their three-dimensional crosslinked network structure, prolonged tissue retention and highly hydrated biocompatible matrices at the location of infection. Zambom et al. constructed a liposomal system containing Histatin 5 analogs in order to treat oral candidiasis (Zambom et al., 2019). The system demonstrated sustained release over 96 h and maintained antifungal activity against *Candida albicans in vitro* over the course of 72 h, which prolonged the antifungal activity of the peptide (Zambom et al., 2019). Also, responsive hydrogels can function as on-demand antifungal delivery systems. Vera-González et al. further demonstrated that enzyme-responsive hydrogels enabled infection-triggered antifungal release with favorable biocompatibility. When exposed to *Candida albicans* secreted enzymes, the hydrogel scaffold undergoes targeted degradation, releasing the drug and enabling infection-responsive drug delivery at infection sites (Vera-Gonzalez et al., 2024). The system demonstrated effective antifungal activity in *in vitro* models and was not toxic to mammalian cells.

PLGA is an FDA-approved biodegradable polymer widely used in drug delivery systems and has found many applications in the construction of micro/nano delivery systems. Its hydrolysis in the body metabolism leads to the formation of lactic and glycolic acids, which are biocompatible and possess considerable translational potential. The use of encapsulation and controlled-release strategies in PLGA nanoparticles may help address major translational limitations of AFPs, including rapid degradation, short half-life, and low bioavailability. Such strategies include decreased access to bodily proteases, increased local retention time, and constant drug delivery. Furthermore, PLGA nanoplatforms have been applied to treat drug-resistant fungi and improve tissue penetration and antibiofilm activity of standard antifungal drugs. As an example, Yang et al. packed AmBisome particles on PLGA nanoparticles and used them together with low-energy low-dose ultrasound treatment, which significantly improved inhibition/elimination of *Candida albicans* biofilms (Yang et al., 2019). The findings indicate the engineering advantages of PLGA nanodelivery during drug-resistant biofilm-associated infections. Additionally, Kolge et al. prepared chitosan-PLGA composite nanocarriers to deliver fluconazole, which showed greater sustained-release and therapeutic efficacy under acidic conditions (Kolge et al., 2023). The system demonstrated lower MIC values and improved safety profiles against resistant *Candida albicans* and *Candida auris*, suggesting that polymeric nanodelivery represents a feasible strategy to achieve improved antifungal efficacy while reducing systemic toxicity (Kolge et al., 2023). In peptide delivery applications, PLGA nanoparticles have strong peptide encapsulation capability and allow sustained release. As an example, Water et al. established that PLGA nanoparticles exhibit high peptide encapsulation efficiency and favorable pharmacodynamic performance in infection models and provided evidence supporting the suitability of PLGA as a peptide delivery system (Water et al., 2015). Analogously, the lung delivery study of AMPs by Casciaro et al. demonstrated that PLGA nanoparticles improve peptide transport through complex mucus barriers, and prolong therapeutic activity *in vivo* (Casciaro et al., 2019). These findings provide a reference for applying similar delivery strategies to treatments of airways or focal infections based on the use of AMPs.

Due to its biodegradability, mucoadhesive properties, positive surface charge, and ease of ion cross-linking into gels/pellets, chitosan is a good candidate material in the area of local delivery of AFPs. In addition, chitosan itself has antifungal activity against fungi including *Candida* species by interacting with negatively charged fungal cell surface components, thereby disrupting cell wall/membrane integrity and fungal tolerance mechanisms. As an example, Shih et al. showed in a *Candida albicans* model that chitosan has antifungal properties by modulating transcriptional networks associated with cell surface integrity, providing mechanistic evidence supporting carrier–drug synergy (Shih et al., 2019). In contrast, chitosan nanoparticles or complex carriers offer advantages in the case of biofilm-related infections. Studies conducted by Gondim et al. showed that chitosan nanoparticles could inhibit biofilm formation and decrease fungal burden on denture base materials, suggesting potential applications in oral sustained-release and infection-prevention systems (Gondim et al., 2018). Even more importantly, experimental works have been published showing both enhanced AFP delivery and antifungal efficacy within chitosan systems. Torres-Rêgo et al. designed degradable chitosan nanoparticles cross-linked using TistH, a peptide derived from scorpion venom gland extracts (Torres-Rego et al., 2019). It was found that this system at the nanoscale had high antimicrobial activity against various species of *Candida* and enhanced antibiofilm activity, thus indicating the direct benefits of chitosan nanodelivery in enhancing AFP stability and anti-biofilm efficacy (Torres-Rego et al., 2019). Also, chitosan may be used with polymers, such as PLGA, to produce composite nanocarriers. These systems exhibit positive surface charge and enhanced binding to fungal cell walls/membranes and pH-dependent release which gives more efficacy and less systemic toxicity when used to treat drug resistant fungi (Kolge et al., 2023). Consequently, within the translational framework discussed in this review, chitosan carriers are not just a material means of local AFP delivery but they might also provide dual carrier and antifungal functions to candidate peptides through self-induced antifungal and anti-biofilm actions.

### 6.3 Manufacturing and regulatory considerations

The industrial-scale manufacturing cost of AFPs remains a major barrier to clinical translation. In contrast to small-molecule antifungal agents, peptides are typically synthesized using solid-phase peptide synthesis (SPPS). As purification complexity and production costs rise substantially when handling peptide candidates with longer sequences, rich in hydrophobic residues, or with more than two disulfide bonds, coupling efficiency decreases, and the generation of deletion peptides and oxidation byproducts increases. Isidro-Llobet et al. have systematically analyzed the use of solvents, yield losses and waste disposal issues in peptide synthesis between R&D and scale-up, emphasizing that process complexity increases substantially during GMP-compliant scale-up, increasing both economic and environmental burdens (Isidro-Llobet et al., 2019). Moreover, in AFP development, numerous AFP candidates are structurally optimized in order to improve their *in vivo* stability. Although this enhances pharmacokinetic behavior, these modifications are often synthetically challenging and difficult to analyze. Zane et al. state that peptide drug development should balance functional improvement and manufacturability so as to avoid unmanageable chemistry, manufacturing, and control (CMC) risks and costs at a later stage (Zane et al., 2021). Hence, incorporating manufacturability assessment during computational prioritization may improve translational success rates from *in vitro* screening to industrial-scale production.

Regulatory evaluation of AFPs for multidrug-resistant fungal infections faces two major challenges: first, uncertainty associated with novel molecular entities (NMEs); second, increased regulatory complexity arising from advanced delivery systems. With the conjugation of AFPs with carriers, including liposomes, PLGA, chitosan, or hydrogels, AFP formulations evolve from simple active substances into complex nano- or local-delivery systems. Critical quality attributes (CQAs) include peptide purity, structural integrity, particle size distribution, encapsulation efficiency, release profiles, and long-term stability. Desai observes that the nanomedicine translation challenge does not necessarily lie in the initial efficacy but in the incomplete understanding of structure–process–performance relationships thereby increasing regulatory uncertainty (Desai, 2012). In addition, the need to have consistent regulatory requirements regarding characterization depth and comparability has been highlighted by Bremer-Hoffmann et al. (2018). Systematic physicochemical and biological characterization of complex formulations is especially required to achieve batch-to-batch consistency and clinical reproducibility (Bremer-Hoffmann et al., 2018). Therefore, within the proposed computational prioritization and experimental validation framework, standardized analytical models should be implemented and efficacy correlation metrics, including translational relevance to MIC and biofilm inhibition models, should be introduced earlier in order to minimize the need for additional studies after entry into the IND phase.

AFPs typically induce rapid membrane disruption under *in vitro* conditions, but their amphiphilic and cationic nature can also induce hemolysis and inflammatory activation, both of which represent major safety concerns that should be addressed through a systematic approach to translational development. Mahlapuu et al. observed that further clinical progression of antimicrobial/antifungal peptides frequently is limited by ambiguity concerning hemolytic activity, cytotoxicity, and immunogenicity, which requires the implementation of systematic *in vitro* hemolysis tests, cytokine release evaluation, and animal toxicity tests already at the initial stage (Mahlapuu et al., 2016). In the context of multidrug-resistant fungal infections, appropriate selection of preclinical models is critical. Planktonic cell models may not accurately predict clinical efficacy of these compounds in biofilm-associated resistant infections. Thus, combined evaluation using biofilm models and immunocompromised animal models is recommended to improve the translational predictability. Moreover, when AFPs are delivered using nanodelivery systems, evaluation of carrier toxicity and tissue accumulation, and long-term safety of degradation products should be performed in order to satisfy regulatory requirements for comprehensive safety evaluation of combination formulations (Ragelle et al., 2017).

### 6.4 Future perspectives: positioning of AFPs in the management of drug-resistant mycoses

Due to the increasing global burden of multidrug-resistant fungal infections, especially infections caused by *Candida auris* and azole-resistant *Candida albicans* and triazole-resistant *Aspergillus fumigatus*, there is an urgent need to develop antifungal agents with alternative mechanisms of action. The latest epidemiological and mechanistic investigations indicate the high rate of development of antifungal resistance as well as the small increase in the number of antifungal drugs under development (Fisher et al., 2022). In this situation, AFPs are an example of a mechanistically dissimilar class of drugs that will require appropriate pharmacological and translational alignment before they can be incorporated into antifungal therapy.

Antifungal resistance is often associated with ERG11 mutations, increased expression of efflux transporters, changes in stress-responsive pathways and tolerance due to biofilms (Arastehfar et al., 2020). By contrast, most AFPs exert antifungal activity through membrane disruption, induction of oxidative stress, disruption of intracellular homeostasis, as well as having immunoregulatory properties. This multimodal mechanism of action reduces dependence on single molecular targets and has been associated with a lower likelihood of stable resistance development. Recent studies indicate that membrane-active peptides retain antifungal activity against isolates displaying classical mechanisms of azole resistance, and this indicates that the direct disruption of fungal lipid bilayers could bypass classical resistance processes. These pharmacological mechanistic differences support the integration of AFPs into therapeutic strategies targeting drug-resistant fungal infections (Mahlapuu et al., 2016).

Extracellular matrix barriers, stress response regulation, and the development of persister cells substantially reduce fungal biofilm susceptibility to antifungal agents (Kaur and Nobile, 2023). Antifungal drugs are more effective when membrane-active peptides are used to disrupt biofilm architecture and increase drug penetration. As an example, Galdiero et al. showed that the membrane-affinity peptide gH625 eliminates persister cells in *Candida albicans* biofilms and enhances the efficacy of conventional antifungal therapies (Galdiero et al., 2020). In the same manner, Sun et al. indicated that AMP-17 effectively inhibits and destroys *Candida albicans* biofilm structures, suggesting that AMPs play dual roles in biofilm-associated infections, through both direct antifungal activity and biofilm disruption (Sun et al., 2022). However, AFPs often exhibit limited *in vivo* stability and exhibit narrow therapeutic windows due to their protease degradation, renal clearance, and non-specific tissue distribution in conditions of systemic administration. As a result, nanodelivery systems have emerged as critical strategies for peptide drug translation in recent years (Younis et al., 2022). Controllable release properties and high biocompatibility make degradable polymeric nanocarriers like PLGA popular in peptide drug delivery studies (Stevanovic et al., 2025). Furthermore, nanomedicines have major challenges in clinical translation, including quality control, prediction of *in vivo* biodistribution, and batch-to-batch consistency (Csoka et al., 2021). Therefore, AFP development strategies should incorporate delivery systems at the molecular design stage to establish integrated molecule–carrier–release systems rather than developing active peptides in isolation.

In addition to delivery and formulation challenges, the large-scale manufacturing of AFPs also raises concerns regarding production cost, solvent consumption, and environmental sustainability associated with conventional solid-phase peptide synthesis (SPPS). Recent advances in green peptide chemistry have introduced safer and more sustainable peptide manufacturing strategies, including the use of green solvents, alternative resin-compatible synthesis systems, and environmentally friendly deprotection approaches for SPPS (Noki et al., 2026; Vivenzio et al., 2025). These emerging approaches may reduce hazardous chemical waste while improving manufacturing sustainability and scalability, thereby facilitating the future industrial production and clinical translation of AFP-based therapeutics.

Peptide therapeutics have unique manufacturing and regulatory challenges, such as synthetic complexity, impurity profiling, scalability, and lot-to-lot reproducibility. Recent studies emphasize the importance of early-stage developability assessment because late-stage translational failure may become a serious problem in the future (Lau and Dunn, 2018). Regulatory assessment with respect to nanoformulated peptide products covers more than just the molecular characterization with regard to nanoparticle physicochemical characterization, release kinetics, stability, and other reproducibility measures. A recent regulatory science literature emphasizes the need to define precise CQAs and structure–property relationships to reduce ambiguity throughout the clinical translation process (Younis et al., 2022). Such characterization frameworks are particularly important in complex peptide–nanocarrier systems when minor changes to the formulation parameters can cause significant changes to *in vivo* behavior.

Current evidence suggests that AFPs may be more appropriately integrated into modern antifungal treatment instead of treating them as direct substitutes for existing antifungal agents. Peptides that target membranes are still active against resistant fungi that utilize target-site mutations or overexpression of efflux pumps as their main resistance mechanism (Robbins et al., 2016). In addition, there is an ongoing therapeutic challenge with biofilm-associated infections in *Candida* species that are multidrug resistant. Since AMPs have been found capable of disrupting biofilm architecture and enhancing antifungal penetration, they can be considered to have a mechanistic justification as an add-on treatment in resistant infections (Kaur and Nobile, 2023; Sun et al., 2022).

## 7 Conclusion

Multidrug-resistant fungal infections have become a major challenge in antifungal therapy, particularly infections caused by *Candida auris*, azole-resistant *Candida albicans*, and triazole-resistant *Aspergillus fumigatus*. The 2022 WHO Fungal Priority Pathogen List further highlights the global threat posed by resistant fungal pathogens (WHO, 2022). Currently available antifungal drugs mainly target a limited number of pathways, such as ergosterol biosynthesis and β-1,3-glucan synthesis. Resistance development is associated with mechanisms such as efflux pump activation, target modification, and biofilm formation (Hoenigl et al., 2021). Therefore, the development of antifungal agents with alternative mechanisms of action remains an important research priority.

AFPs offer several advantages in this context. Due to their cationic and amphiphilic structural properties, they may exert antifungal activity through membrane targeting, inducing oxidative stress, disrupting intracellular homeostasis, and altering the structure of biofilms. AFPs are less likely to induce stable resistance due to their multi-target mechanisms (Fernandez de Ullivarri et al., 2020). Moreover, biofilm-associated resistance is considered a major contributor to antifungal treatment failure. AFPs can disrupt biofilm matrices and enhance the efficacy of conventional antifungal drugs, supporting their potential use in combination therapies (Kaur and Nobile, 2023). This review highlights that AFP development should follow a systematic strategy involving sequence mining, motif identification, machine learning-based screening, molecular modeling, and experimental validation. Recent studies on peptide therapeutic development suggest that early consideration of manufacturability and stability may reduce late-stage translational failure and improve clinical development efficiency (Wang et al., 2022). Nonetheless, the clinical development of AFPs still faces challenges related to stability, hemolytic toxicity, immunogenicity and delivery system complexity. The short *in vivo* half-life and susceptibility of peptide molecules to protease degradation constrain their potential applications in systemic therapy. In addition, the cationic nature of AFPs may complicate lipid nanoparticle encapsulation and delivery because many currently established nanodelivery systems were originally optimized for negatively charged nucleic acid cargos such as DNA or RNA. These findings suggest that, we may need to adopt peptide-specific formulation strategies to enhance loading efficiency, stability, and delivery performance. Thus, the engineering of AFP structures and delivery systems (liposomes, PLGA, chitosan, hydrogels) should be integrated early into molecular design strategies in the development of AFP, rather than being considered only during later stages of development (Puumala et al., 2024).

Research on AFPs has progressed from basic investigations of their structure, function, and mechanisms of action. As complements to existing antifungal agents, it is expected that AFPs may become part of combination therapies and provide an alternative mechanism of action as antifungal agents. The membrane and biofilm disruptive mechanism of AFPs may be especially useful in treating drug resistant fungal infections. The construction of an integrated development pipeline incorporating computational predictions, structural refinement, laboratory testing, and strategic incorporation of purposeful and engineered delivery systems, is the next step to enhancing the translatability and the clinical applicability of AFPs. With advances in AI-assisted design, nanodelivery systems, and standardized infection models, AFPs are expected to play an increasingly important role in antifungal therapy. AFPs are likely to occupy a more defined and sustainable position in the management of drug-resistant fungal infections.
